# Cardiac Atrial Appendage Stem Cells Embedded in Elastin-like Recombinamer Hydrogel for Cardiac Repair After Acute MI: A Pilot Study

**DOI:** 10.3390/ijms27219563

**Published:** 2026-10-26

**Authors:** Ellen Heeren, Lotte Vastmans, Dorien Deluyker, Anouk Delaet, Lisa Steegen, Vincent Vandenboer, Ignace Van der Wee, María Puertas Bartolomé, Sam Duwé, Sebastiaan Dhont, Pieter-Jan Volders, Alessio Cardilli, José Carlos Rodríguez-Cabello, Annelies Bronckaers, Marc Hendrikx, Virginie Bito

**Affiliations:** 1 Cardio & Organ Systems (COST), Biomedical Research Institute (BIOMED), Hasselt University, Agoralaan, 3590 Diepenbeek, Belgium; ellen.heeren@uhasselt.be (E.H.); lotte.vastmans@uhasselt.be (L.V.); dorien.deluyker@uhasselt.be (D.D.); anouk.delaet@uhasselt.be (A.D.); lisa.steegen@uclouvain.be (L.S.); v.vandenboer@gmail.com (V.V.); ignacevanderwee@gmail.com (I.V.d.W.); annelies.bronckaers@uhasselt.be (A.B.); mjh.hendrikx@gmail.com (M.H.); 2 BIOFORGE (Group for Advanced Materials and Nanobiotechnology), University of Valladolid—CIBER-BBN-LADIS, Edificio Lucía, Paseo de Belén 19, 47011 Valladolid, Spain; maria.puertas@uva.es (M.P.B.); roca@bioforge.uva.es (J.C.R.-C.); 3 Advanced Optical Microscopy Centre, Biomedical Research Institute (BIOMED, Hasselt University, Agoralaan, 3590 Diepenbeek, Belgium; sam.duwe@uhasselt.be; 4 Limburg Clinical Research Center (LCRC), Hasselt University, 3590 Diepenbeek, Belgium; sebastiaan.dhont@zol.be (S.D.); pieter-jan.volders@jessazh.be (P.-J.V.); 5 Department of Immunology and Infection, Biomedical Research Institute (BIOMED), Hasselt University, Agoralaan, 3590 Diepenbeek, Belgium; alessio.cardilli@uhasselt.be

**Keywords:** cardiac progenitor cells, ELR hydrogel, myocardial infarction, cardiac repair

## Abstract

Myocardial infarction (MI) causes extensive cardiomyocyte loss and adverse remodeling, frequently progressing to heart failure. Although stem cell-based therapies are being investigated for cardiac repair, clinical outcomes remain modest. Cardiac atrial appendage stem cells (CASCs) harbor cardiomyogenic potential, making them promising candidates for cardiac regeneration. This proof-of-concept pilot study investigates the feasibility and preliminary therapeutic potential of delivering CASCs within an elastin-like recombinamer (ELR) hydrogel scaffold following MI. MI was induced in rats by permanent ligation of the left anterior descending coronary artery. Immediately after occlusion, CASCs (MI+C), ELR hydrogel (MI+H), or CASCs embedded in ELR hydrogel (MI+C+H) were injected intramyocardially into the peri-infarct region. MI and SHAM-operated animals served as controls. Four weeks post-MI, echocardiography showed a large, non-significant left ventricular ejection fraction improvement in MI+C+H versus MI, with a trend toward reduced end-diastolic volume. MI+C+H significantly improved anterior wall thickness versus MI+H, with favorable trends for MI+C. Wall motion score index showed a similar large, non-significant effect. Transcriptomics suggested the MI+C+H molecular profile reflected complementary contributions of both monotherapies rather than distinct synergistic mechanisms. These preliminary findings support ELR-mediated CASC delivery and suggest potential benefits for preserving cardiac function and limiting adverse remodeling, warranting confirmation in adequately powered studies.

## 1. Introduction

Despite major advances in prevention and reperfusion therapies, ischemic heart disease (IHD) remains the leading cause of death worldwide, accounting for approximately 13% of global mortality in 2021 [1]. Among its clinical manifestations, myocardial infarction (MI) is one of the most severe, leading to significant cardiomyocyte loss and can ultimately progress to heart failure (HF) if left untreated [2]. While conventional therapies for MI have significantly improved outcomes, they primarily aim to restore coronary perfusion rather than replace lost myocardium, highlighting the urgent need for regenerative approaches to restore cardiac tissue and prevent progression to HF [3].

Efforts to regenerate the injured myocardium have placed stem cell-based therapies at the forefront of cardiovascular research [4]. Numerous preclinical and clinical studies have investigated the regenerative potential of bone marrow–derived mononuclear cells (BM-MNCs), mesenchymal stem cells (MSCs), and induced pluripotent stem cells (iPSCs), among others [5]. Although the underlying mechanisms remain incompletely understood, growing evidence indicates that the benefits of BM-MNCs and MSCs are mediated through paracrine signaling rather than true cardiomyogenic differentiation [6,7,8]. In contrast, iPSCs have demonstrated significant cardiomyogenic potential, but their tumorigenic risk and immature electrophysiological phenotype with associated arrhythmia risk limit their translational application [9]. Cardiac progenitor cells (CPCs) are an attractive alternative as they are inherently predisposed toward the cardiac lineage [5]. Although paracrine signaling is considered the primary driver of the CPC’s therapeutic capacity, cardiac atrial appendage stem cells (CASCs) have demonstrated a superior cardiomyogenic differentiation potential both in vitro and in vivo [10,11]. Additionally, CASCs have been shown to promote angiogenesis via paracrine mechanisms both in vitro and in vivo [12]. Following transplantation in a porcine model of MI, CASCs have been shown to functionally integrate into the host myocardium, preserving left ventricular ejection fraction (LVEF) and decreasing scar size [11,12,13]. Together, these properties position CASCs as a suitable candidate for myocardial repair following MI. Nevertheless, regardless of their therapeutic potential, major challenges shared by all stem cell-based therapies remain the poor survival, retention, and engraftment of transplanted cells in the hostile post-infarct environment [14]. The post-MI myocardium is characterized by inflammation, oxidative stress, hypoxia, and disrupted extracellular matrix [15]. Effective engraftment and survival of transplanted cells depend on an intact ECM that supports fibronectin-integrin interactions, which are essential for cell adhesion and activation of intracellular survival pathways. Disruption of these interactions in the infarcted myocardium leads to loss of integrin-mediated signaling, which can trigger anoikis, a form of apoptosis induced by cell detachment, thereby contributing substantially to the rapid loss of transplanted cells after delivery [16].

To overcome low cell survival and retention rates associated with anoikis and the hostile post-infarct environment, bioactive scaffolds have been developed to provide structural support and pro-survival signals to transplanted cells [17]. The elastin-like recombinamer (ELR) hydrogel mimics the ECM. Its modular and customizable design allows tailoring to specific applications. In the present study, the hydrogel incorporates the arginine-glycine-aspartate motif, a fibronectin-derived sequence known to promote cell adhesion through integrin binding [18]. In addition, the modified hydrogel displayed low immunogenicity, and its thermoresponsive behavior enables intramyocardial injection. Importantly, the current ELR-hydrogel has been proven to be biocompatible with MSCs, supporting general biocompatibility with other stem cells [19]. In an ovine model of MI, subacute delivery (7 days post-MI) of an ELR hydrogel combined with MSCs improved LVEF, reduced fibrosis, and stimulated angiogenesis [20].

Based on the complementary properties of CASCs and the ECM-mimetic features of the ELR hydrogel, the present proof-of-concept pilot study evaluated the feasibility of ELR hydrogel-mediated delivery of CASCs in a rat model of MI and explored its preliminary effects on cardiac repair and the underlying molecular responses.

## 2. Results

### 2.1. ELR Hydrogel Supports CASCs Viability

**Figure 1:**
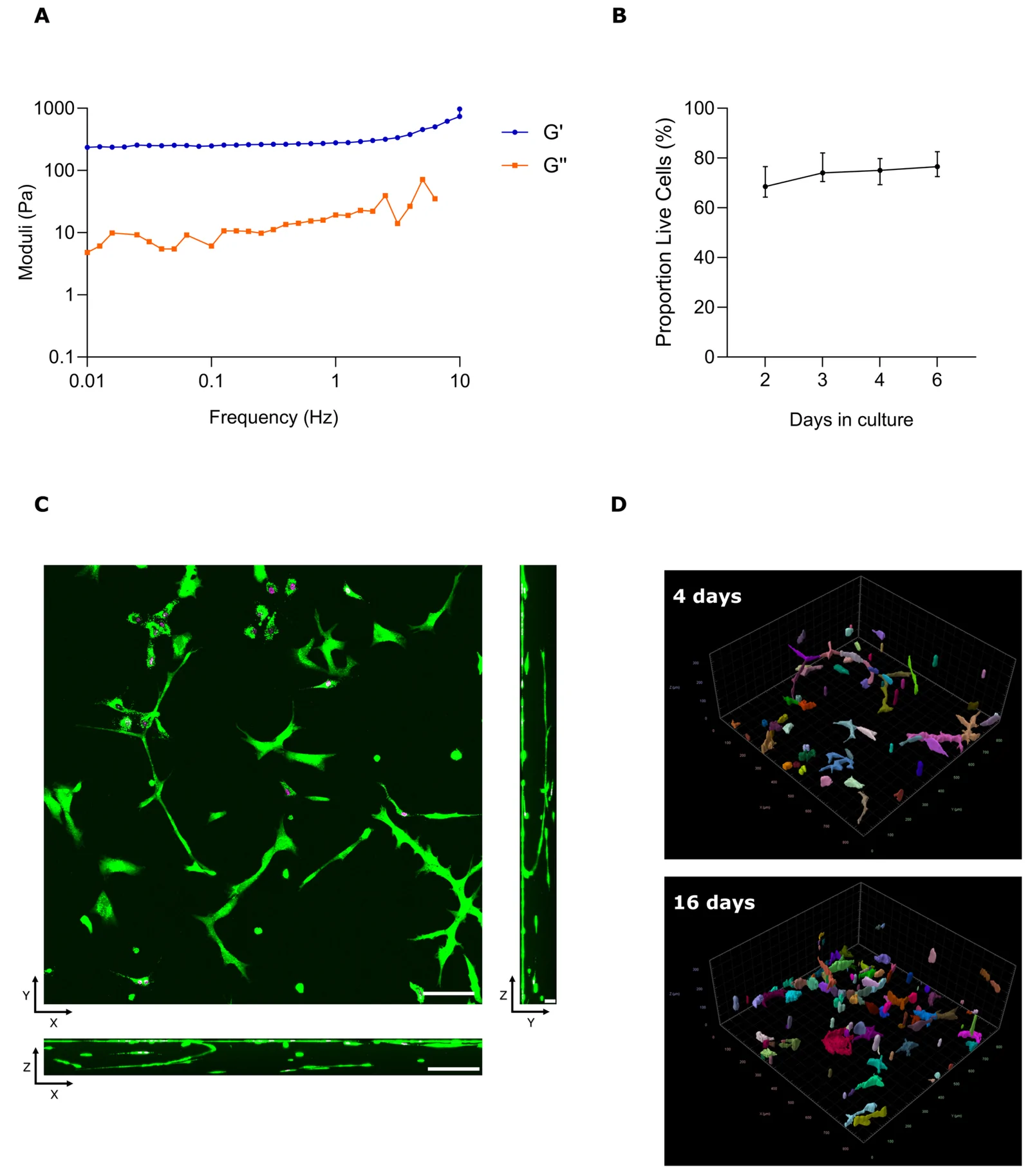
Characterization of the ELR Hydrogel and CASCs Viability. (**A**) Frequency-sweep rheology of the ELR hydrogel showing storage modulus (G′) and loss modulus (G″) between 0.01 and 10 Hz, confirming predominantly elastic behavior. (**B**) Viability of CASCs embedded in the hydrogel at days 2, 3, 4, and 6 post-seeding, quantified using LIVE/DEAD staining. Data are presented as median and interquartile range (IQR) (*n* = 6). No significant differences were observed between groups (Kruskal–Wallis test with Dunn’s correction). (**C**) Maximum-intensity projection images at day 4 post-seeding in the XY, YZ, and XZ planes. Live cells are shown in green (Calcein-AM), and dead cells in magenta (Ethidium homodimer-1). White scale bars = 100 µm. (**D**) Volumetric 3D renderings of segmented CASCs at 4 and 16 days post-seeding, illustrating sustained cellular presence throughout the hydrogel.

To assess the viscoelastic properties of the ELR hydrogel, rheological frequency-sweep measurements were performed over a range of 0.01–10 Hz. The hydrogel formed a soft, predominantly elastic network, as evidenced by the storage modulus (G′) consistently exceeding the loss modulus (G″) across the tested range, indicative of solid-like behavior and the presence of a defined polymer network. Under fully crosslinked conditions, the hydrogel displayed a storage modulus of approximately 250 Pa within the linear viscoelastic region, consistent with a soft hydrogel (Figure 1A). The crosslinking kinetics provided handling characteristics compatible with clinical application, allowing smooth injection into the peri-infarct region. CASCs encapsulated within the hydrogel remained viable throughout culture, as shown by LIVE/DEAD staining (day 2: 69.8 ± 6.7%, day 3: 75.5 ± 6.2%, day 4: 75.0 ± 6.3%, day 6: 77.2 ± 5.0%; Figure 1B). No decline in viability was observed over time, indicating that the gel provides a permissive and non-cytotoxic environment for CASCs. Maximum intensity projections demonstrated a uniform distribution of CASCs within the hydrogel with elongated, spindle-like morphologies typical of cells embedded in a 3D matrix (Figure 1C). The volumetric 3D renderings further confirmed the spatial organization of individual cells throughout the entire gel volume (Figure 1D). Segmented reconstructions at days 4 and 16 of cell culture revealed clearly defined cell bodies, with an increased number of segmented objects at the later timepoint, suggesting persistent cellular presence over time, although cell proliferation was not quantitatively assessed.

### 2.2. CASCs-ELR Hydrogel Treatment Effects on Global and Regional Cardiac Function

To evaluate whether MI and subsequent cell-based interventions affected cardiac function, invasive LV hemodynamic measurements were performed four weeks post-surgery. LV systolic pressure (LVSP), LV end-diastolic pressure (LVEDP), maximum rate of pressure rise (dP/dt_max_), maximum rate of pressure fall (dP/dt_min_), and the time constant of LV relaxation (Tau) were comparable across all experimental groups (Table 1). No significant differences or trends were observed between SHAM and MI animals, indicating that at four weeks post-intervention, resting invasive hemodynamic function was not measurably altered by MI. Similarly, none of the treatment groups differed from MI after Holm–Bonferroni correction. Collectively, these findings indicate that this pilot study did not detect measurable differences in baseline invasive hemodynamic parameters between experimental groups.

Successful MI induction was confirmed by marked changes in LV function and remodeling in MI compared with SHAM animals (Supplementary Figure S1). MI was associated with a significant reduction in LVEF, increased end-diastolic volume (EDV) and end-systolic volume (ESV), and pronounced thinning with impaired systolic thickening of the anterior wall, consistent with the development of post-MI systolic dysfunction and adverse LV remodeling (*p* < 0.05).

**Figure 2:**
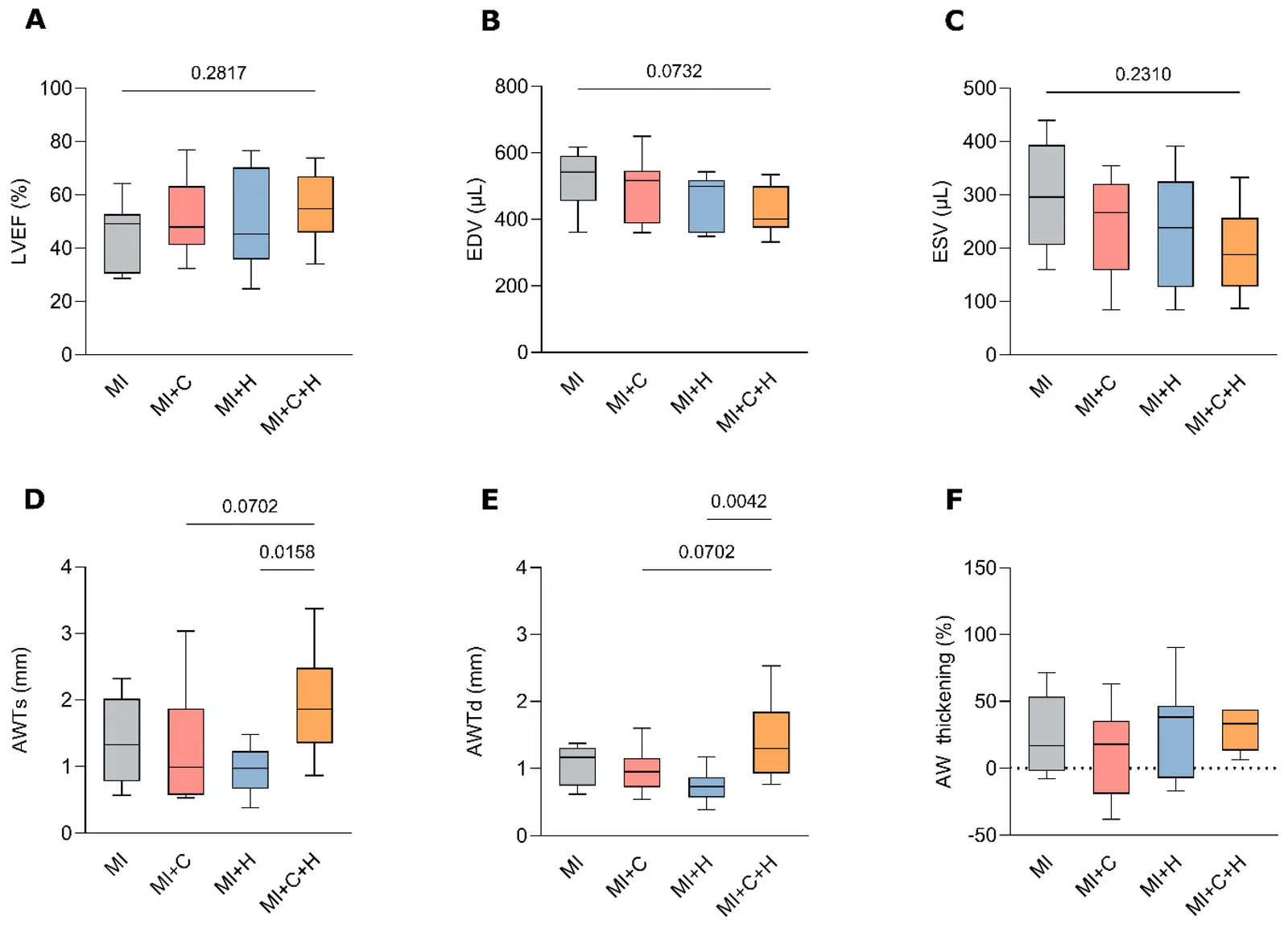
Echocardiographic assessment of global LV function and structural remodeling following MI and therapeutic interventions. (**A**) Left ventricular ejection fraction (LVEF), (**B**) end-diastolic volume (EDV), (**C**) end-systolic volume (ESV), (**D**) anterior wall thickness in systole (AWTs) and (**E**) diastole (AWTd), and (**F**) anterior wall (AW) thickening (%) were assessed by echocardiography. Sample sizes for LVEF, EDV, and ESV were; SHAM: *n* = 7, MI: *n* = 9, MI+C: *n* = 10, MI+H: *n* = 9, and MI+C+H: *n* = 9. For AWTs, AWTd, and AW thickening, sample sizes were: SHAM: *n* = 7, MI: *n* = 9, MI+C: *n* = 10, MI+H: *n* = 9, and MI+C+H: *n* = 7. Comparisons were performed using Mann–Whitney tests followed by Holm–Bonferroni correction for MI vs. the therapy groups and for MI+C+H vs. MI+C and MI+H. Data are presented as median (line) and interquartile range (IQR). Whiskers extend to the most extreme values within 1.5 × IQR. Exact *p*-values are indicated on the figure.

We next explored whether the therapeutic interventions influenced global LV function compared with untreated MI animals. After Holm–Bonferroni correction, no statistically significant differences in LVEF were observed among the MI groups. Median LVEF values were 49.1% [30.5–52.7] in MI, 48.0% [41.1–63.2] in MI+C, 45.3% [35.8–70.3] in MI+H, and 54.8% [45.9–66.9] in MI+C+H (Figure 2A). Although not statistically significant, MI+C+H showed the highest improvement relative to MI. To estimate the magnitude of this change, mean difference and effect size based on mean ± SD values were calculated and revealed an increase of +11.2 percentage points compared with MI (95% CI: −1.51 to +23.84), corresponding to a large effect size (Hedges g = 0.84). However, the wide confidence interval indicates considerable uncertainty in the estimated effect, consistent with the small sample size. Analysis of LV volumes did not reveal significant differences in EDV and ESV after correction. Nevertheless, MI+C+H showed a non-significant tendency toward lower EDV compared with MI (189 µL [129–257] vs. 296 µL [207–394]; *p*_adj = 0.0732; Figure 2B). ESV did not differ significantly between groups (*p*_adj > 0.10; Figure 2C), although MI+C+H displayed the lowest median values (188.7 µL [128.6–256.7] vs. MI 295.9 µL [206.6–393.8]) again, in line with the data seen for LVEF and EDV.

Regional wall remodeling was evaluated by measuring anterior wall thickness in systole (AWTs; Figure 2D) and diastole (AWTd; Figure 2E), and anterior wall (AW) thickening (Figure 2F). No significant differences were observed between MI and any treatment group after correction. When comparing treatment modalities, AWTs were significantly higher in MI+C+H than in MI+H (median 1.87 mm [1.35–2.48] vs. 0.97 mm [0.67–1.23]; *p*_adj = 0.0158), while MI+C showed a similar yet non-significant pattern (0.99 mm [0.57–1.87]; *p*_adj = 0.0702). AWTd showed a comparable pattern with greater values in MI+H+C (1.30 mm [0.93–1.85]) compared with MI+H (0.73 mm [0.57–0.87]; *p*_adj = 0.0042), whereas MI+C again showed a non-significant directional difference (0.96 mm [0.73–1.16]; *p*_adj = 0.0702). AW thickening did not differ significantly between groups, although MI+C+H consistently showed the highest median values (33.2% [13.2–43.9]).

Taken together, this proof-of-concept pilot study did not detect statistically significant treatment effects on global or regional LV function compared with MI. Nevertheless, several functional parameters showed favorable trends in the MI+C+H group, providing preliminary support for further investigation in adequately powered studies. To facilitate the design of future confirmatory experiments, a sample size estimation based on the observed LVEF effect is provided in Supplementary Table S1. Full descriptive statistics are provided in Supplementary Table S2. Interobserver variability for LVEF was low, as assessed by Bland–Altman analysis, with a mean bias of −0.34 percentage points and limits of agreement ranging from −4.37 to +3.70, indicating good consistency.

**Figure 3:**
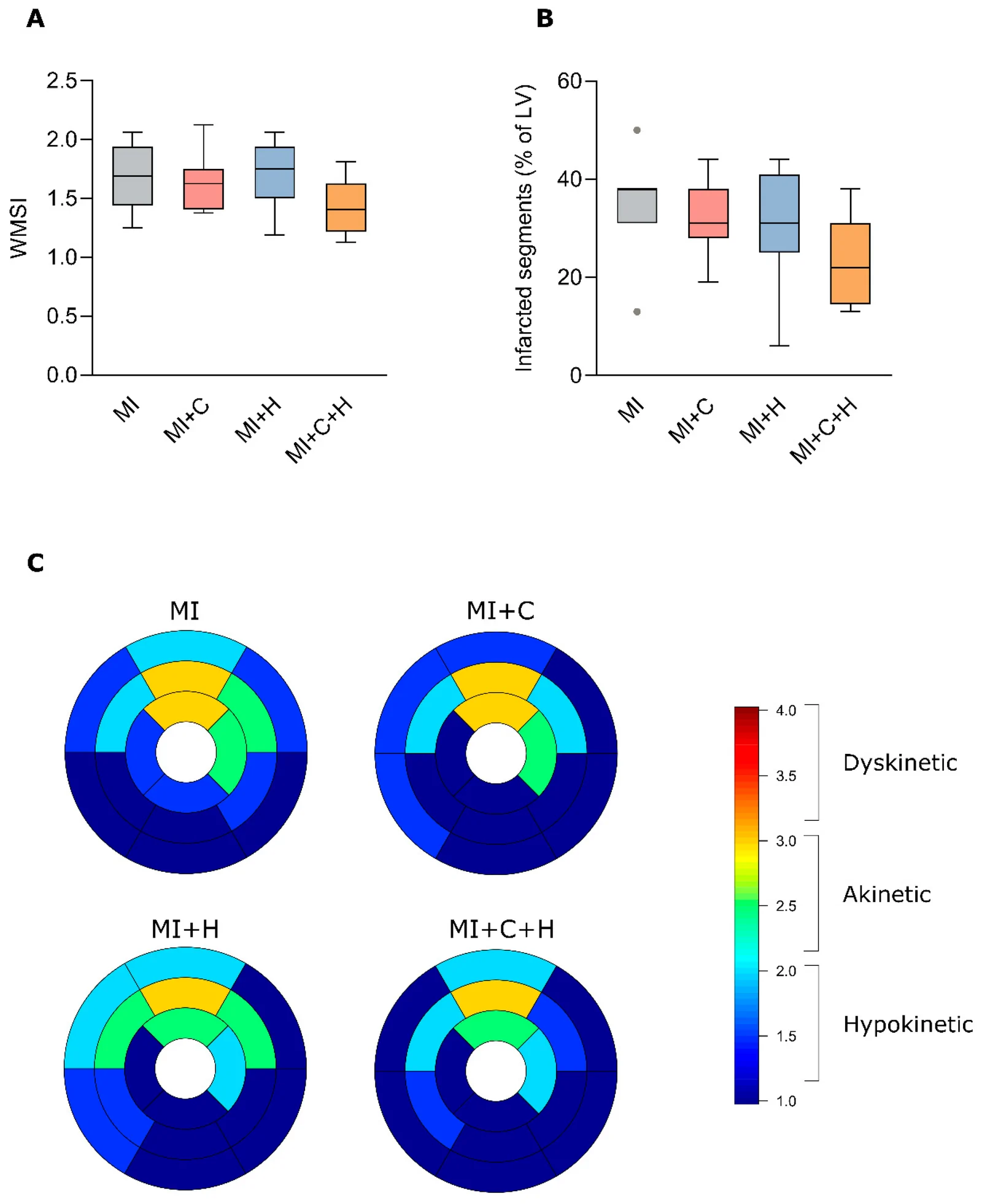
Impact of the different interventions on wall motion score and infarct burden. (**A**) Wall motion score index (WMSI). (**B**) Infarct extent, calculated as the percentage of LV segments with a wall motion score > 2. Sample sizes for WMSI and infarct extent were; SHAM: *n* = 7, MI: *n* = 7, MI+C: *n* = 9, MI+H: *n* = 9, and MI+C+H: *n* = 8. Data are presented as Tukey-style box-and-whisker plots (median, IQR, whiskers = 1.5 × IQR). Values outside the 1.5 × IQR range are displayed as individual outlier points. Comparisons were performed using Mann–Whitney tests with *p* values corrected for multiple comparisons using the Holm–Bonferroni method for MI versus each therapy group, and MI+C+H versus MI+C and MI+H. (**C**) Bull’s eye maps illustrate the spatial distribution of regional dysfunction across short-axis basal, mid-ventricular, and apical levels. Segments are color-coded according to wall motion score: 1 = normal (dark blue), 2 = hypokinetic (light blue to green), 3 = akinetic (yellow to orange), and 4 = dyskinetic (red).

In addition to global echocardiographic parameters, wall motion index (WMSI) was evaluated to assess regional myocardial dysfunction and infarct severity. As shown in Supplementary Figure S2, WMSI was markedly increased in MI compared with SHAM, confirming pronounced regional dysfunction in our model. Among the treatment groups, MI+C+H showed the lowest WMSI (median 1.41 [1.22–1.63]), whereas higher values were observed in MI+C and MI+H (1.63 [1.41–1.75] and 1.75 [1.50–1.94], respectively) (Figure 3A). However, after Holm–Bonferroni correction, none of the treatment groups differed significantly from MI. Quantification of the WMSI difference magnitude between MI+C+H and MI revealed a mean difference of −0.24 (95% CI −0.55 to +0.06), corresponding to a large effect size (Hedges g = 0.86). However, the wide confidence interval reflects the uncertainty inherent to this proof-of-concept pilot study. Wall motion scoring further enabled quantification of infarct size, expressed as the percentage of affected LV segments (Figure 3B). MI animals had a significantly larger infarct size compared with SHAM (Supplementary Figure S2). Among the treatment groups and consistent with previous parameters, MI+C+H showed the lowest median infarct percentage (22% [15–31]), followed by MI+C (31% [28–38]) and MI+H (31% [25–41]). Despite this trend, none of these reductions reached statistical significance after correction for multiple comparisons. The bull’s eye plots summarize these findings which demonstrate clear regional wall motion abnormalities in MI and partial attenuation in the treated groups, particularly in MI+C+H (Figure 3C). Full descriptive statistics are provided in Supplementary Table S3. Finally, interobserver agreement for WMSI was assessed using Bland–Altman analysis. WMSI showed good interobserver agreement, with a near-zero mean bias (0.007) and narrow 95% limits of agreement (−0.25 to +0.26), indicating that the regional wall motion scoring methodology was reproducible between observers.

### 2.3. Associations Between Regional Wall Motion and Global Cardiac Function

**Figure 4:**
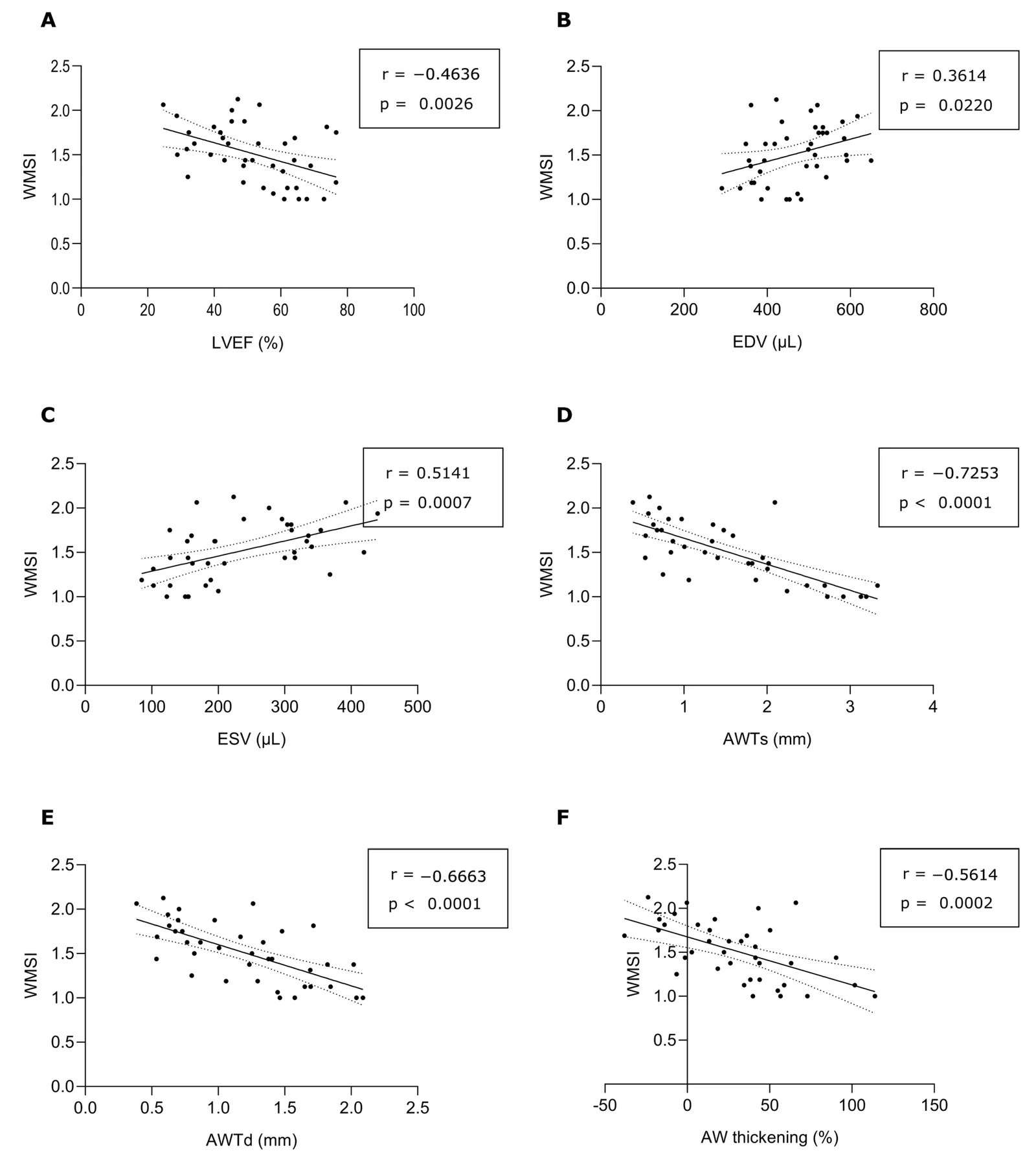
Correlations between WMSI and quantitative echocardiographic parameters 4 weeks post-MI. Scatterplots show the relationships between WMSI and (**A**) left ventricular ejection fraction (LVEF; *n* = 40), (**B**) end-diastolic volume (EDV; *n* = 40), (**C**) end-systolic volume (ESV; *n* = 40), (**D**) anterior wall thickness in systole (AWTs; *n* = 38), (**E**) anterior wall thickness in diastole (AWTd; *n* = 38), and (**F**) anterior wall (AW) thickening (*n* = 38). Spearman correlation coefficients (r) and corresponding two-tailed *p*-values are displayed within each plot. A linear regression line with 95% confidence interval is shown for visualization only. Each data point represents an individual animal.

Considering global echocardiographic parameters and regional wall motion scoring capture complementary aspects of post-MI remodeling, we next explored their interrelationship at the level of individual animals. Correlation analyses were therefore performed between WMSI and the echocardiographic parameters (Figure 4). WMSI showed moderate correlations with global functional parameters, including LVEF (Spearman r = −0.46, *p* = 0.0026) and EDV (r = 0.36, *p* = 0.022), and a slightly stronger correlation with ESV (r = 0.51, *p* = 0.0007). The strongest associations were observed between WMSI and anterior wall measures, namely AWTs (r = −0.73, *p* < 0.0001), AWTd (r = −0.67, *p* < 0.0001), and AW thickening (r = −0.56, *p* = 0.0002). Across all animals, increasing WMSI values were consistently associated with anterior wall thinning, reduced systolic thickening, and ventricular dilation, supporting the use of combined regional and global echocardiographic assessment to characterize post-MI remodeling in this proof-of-concept pilot study.

### 2.4. Molecular Characterization of Treatment Effect

**Figure 5:**
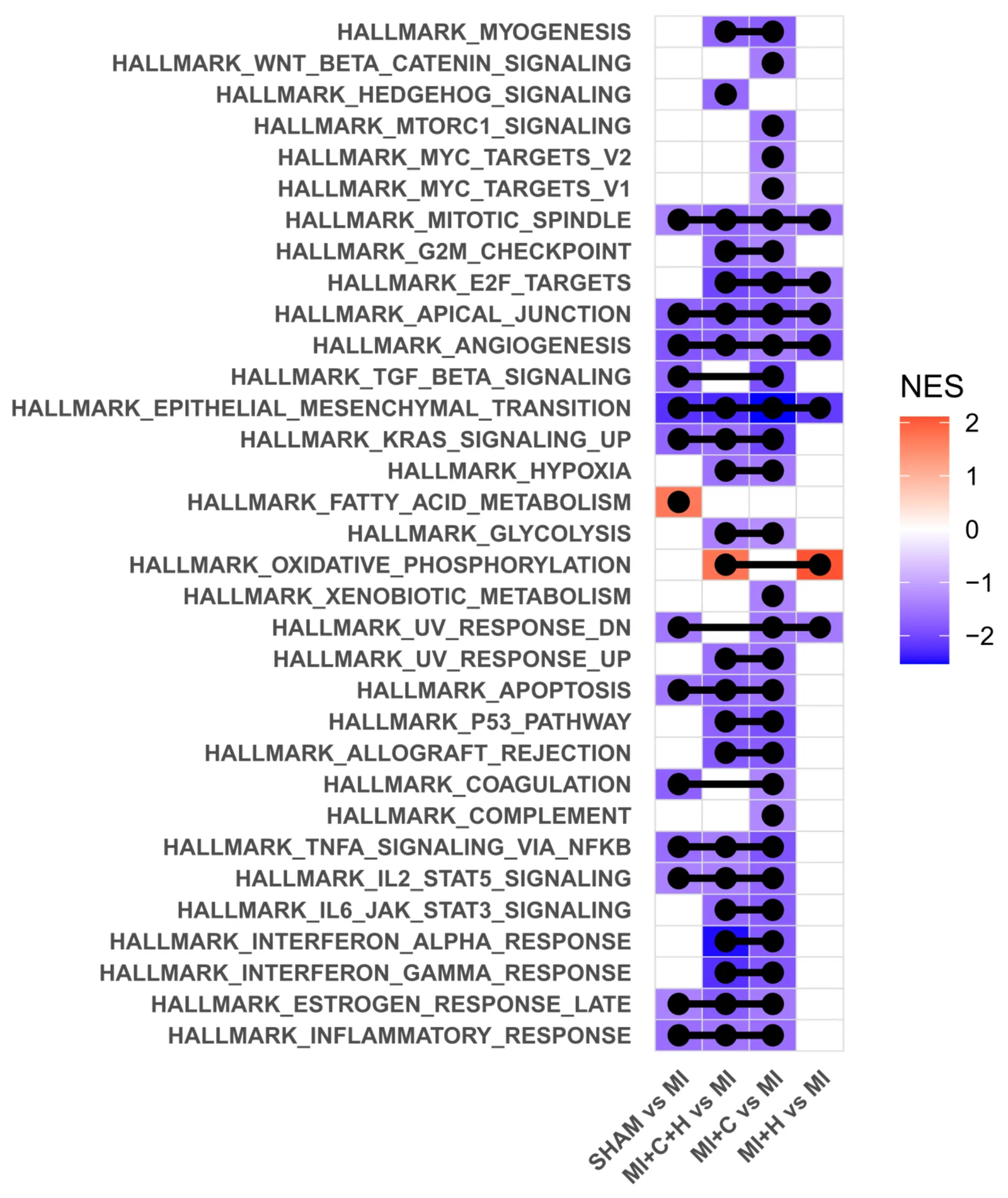
Pathway enrichment profiles across therapies. Gene set enrichment analysis (GSEA) of differential expression profiles using the MSigDB Hallmark collection (50 gene sets). Only significantly enriched/depleted pathways are shown. The heatmap displays normalized enrichment scores (NES) for each therapy group relative to MI. Color indicates the direction and magnitude of enrichment (negative NES = blue, indicating pathway depletion; NES ≈ 0 = white; positive NES = red, indicating pathway enrichment, relative to MI).

To gain insight into the molecular processes underlying the observed functional effects, we next analyzed transcriptomic changes across the respective treatment groups. Considering that individual gene-level differences were modest, we focused on coordinated changes at the pathway level. Gene set enrichment analysis (GSEA) was therefore performed relative to MI, identifying significantly enriched or depleted hallmark pathways (Figure 5). MI hearts showed enrichment of pathways associated with inflammatory signaling, apoptosis, angiogenesis, and extracellular remodeling, alongside depletion of fatty acid metabolism, compared with SHAM hearts. Among the treatment groups, the pathway profile of MI+C+H largely overlapped with those of the monotherapies, especially that of MI+C. Notably, the Hedgehog signaling was the only hallmark uniquely depleted in MI+C+H, whereas all other enriched or depleted pathways were also observed in MI+C, MI+H, or both. This pattern suggests that the molecular effects of the combination therapy reflect complementary contributions of cells and hydrogel rather than a synergistic activation of novel signaling pathways. In terms of individual contributions, MI+C accounted for the broad suppression of inflammatory, proliferative, hypoxic, and remodeling-associated signatures. In contrast, MI+H also showed depleted remodeling-associated pathways but its primary contribution appeared to be metabolic, as reflected by enrichment of the oxidative phosphorylation hallmark. To examine specific genes representing key biological processes relevant to MI, we performed qPCR analysis of predefined inflammatory, remodeling, and oxidative stress-related targets (Supplementary Figure S3). CASCs treatment showed the strongest effect in reducing cellular stress-associated gene expression and inducing antioxidant-associated genes, whereas the combination and hydrogel treatments more prominently attenuated genes related to fibrosis and ECM remodeling. In contrast, several inflammatory genes appeared upregulated following CASCs treatment, highlighting differences between targeted and transcriptome-wide analyses.

## 3. Discussion

Regenerative approaches capable of restoring myocardial structure and function following MI have increasingly shifted towards strategies that combine cellular components with supportive biomaterials [21]. This shift largely reflects the persistent challenge of poor survival following transplantation. In the infarcted myocardium, cells encounter a hostile post-MI environment, characterized by marked inflammation, oxidative stress, and altered ECM, compromising their survival and therapeutic potential. Injectable hydrogels offer a way to mitigate these challenges by providing a protective environment, as well as biomechanical support for the injured myocardium [22]. Within this context, we conducted a proof-of-concept pilot study to evaluate the feasibility and preliminary therapeutic potential of delivering CASCs embedded within an ELR hydrogel in a rat MI model. In this exploratory setting, neither CASCs nor ELR hydrogel monotherapy was associated with detectable improvements in cardiac function compared with MI, whereas the combination therapy showed favorable trends across several global and regional functional parameters. Although the functional findings did not reach statistical significance, several outcomes were accompanied by large effect sizes, suggesting potentially biologically relevant effects that warrant confirmation in adequately powered studies. However, these findings should be interpreted cautiously in the context of the different biomaterial formulations used for CASC delivery in the MI+C and MI+C+H groups. As the present study was not designed to directly compare the two delivery matrices, the observed trends cannot be attributed exclusively to ELR hydrogel or interpreted as definitive evidence that ELR hydrogel enhances CASC efficacy. Further studies using appropriately matched delivery formulations will be required to distinguish the respective contributions of the cells and the biomaterial. Transcriptomic analyses further indicated that the molecular signature of the combination therapy reflected complementary contributions from both monotherapies rather than the emergence of a distinct synergistic transcriptional mechanism.

Recent work has explored the use of peptide and polymer hydrogels as CPC carriers, showing that such scaffolds can promote cell retention and provide functional benefit in rodent MI models [23]. These biomaterials typically share a compliant, predominantly elastic mechanical profile, an important feature, as excessively stiff matrices are known to impair viability and limit engraftment [24]. The ELR hydrogel used in this study fits within this framework, displaying storage moduli in the low hundreds of Pascals. Although such properties make a direct contribution to structural and mechanical reinforcement to the infarcted wall unlikely, they do support its role as a permissive ECM-mimetic scaffold for CASCs delivery. The biocompatibility of ELR hydrogels has been demonstrated previously with MSCs and was reaffirmed in our study through preserved CASCs viability after encapsulation [19].

An important rationale for combining CASCs with an RGD-functionalized ELR hydrogel is its potential to support cell–matrix interactions following transplantation. In the infarcted myocardium, loss of integrin-mediated ECM attachment triggers anoikis, contributing to poor survival of transplanted cells [25]. By providing RGD motifs for integrin engagement and a supportive three-dimensional matrix, the ELR hydrogel may enhance CASCs’ persistence in vivo [26]. However, cell survival and retention were not directly assessed in this study. The observed functional improvements do not establish whether these mechanisms contribute to the outcomes, and dedicated cell-tracking experiments are therefore required to further investigate this possibility.

To assess the effect of the combination therapy on cardiac function, invasive hemodynamic measurements and echocardiography were employed. Invasive hemodynamic measurements are a well-established technique to evaluate LV contractility and relaxation in small animal models of MI [27]. In our study, we did not detect significant differences in hemodynamic parameters between MI and therapy groups, nor between SHAM and MI animals at the four-week timepoint. While pressure measurements are an established method, they may be relatively insensitive to regional dysfunction or potentially overestimate cardiac function [28]. This limitation is reflected in the present dataset, where echocardiographic parameters and wall motion metrics clearly demonstrated post-MI systolic dysfunction, ventricular dilation, and anterior wall thinning in the absence of significant differences in invasive parameters. Although invasive pressure measurements require precise calibration and are inherently sensitive to experimental conditions, all recordings were performed using standardized procedures, making technical variability an unlikely explanation for the observed findings. Together, these findings highlight the complementary nature of invasive and imaging-based assessments in capturing and understanding the complexity of post-MI remodeling.

In contrast to prior rat studies from our group, CASCs monotherapy only modestly modulated cardiac function here. A likely explanation relates to infarct severity. Compared to the previous studies, MI rats in the present cohort exhibited substantially greater functional impairment and more pronounced anterior wall thinning, indicating a more advanced remodeling state [29]. The therapeutic efficacy of cell-based interventions is known to depend on infarct magnitude, inflammatory burden, and ECM composition [15,30,31]. Accordingly, differences in infarct severity likely underlie the discrepancy between the prior findings of Evens et al. and the more modest effects of the CASCs monotherapy observed here. The limited functional recovery with CASCs monotherapy in our study aligns with the broader concept that cell therapy alone may be insufficient to meaningfully prevent remodeling in more severe MI [32].

Similarly, the ELR-hydrogel alone failed to elicit measurable functional recovery in this severe MI setting. This contrasts with previous findings in a non-transmural MI ovine model where the hydrogel injection seven days post-injury decreased fibrosis, promoted angiogenesis, and activated pro-survival pathways [20]. In the study by Contessotto et al., molecular analysis revealed activation of key pro-survival pathways (PI3K/Akt/mTOR), consistent with a mechanism in which the hydrogel protects and stabilizes salvageable ischemic myocardium [20]. Differences in infarct severity, species-specific healing responses, and timing of hydrogel delivery likely account for this discrepancy. In the present study, the severe infarct burden in our cohort is consistent with extensive, likely transmural infarctions, leaving limited salvageable myocardium for such biological rescue mechanisms to act upon. In addition, injections in the present study occurred immediately after LAD ligation, at a time when the inflammatory response is the most pronounced and infarct boundaries are not yet clearly identifiable. Delivery timing is increasingly recognized as a critical determinant of biomaterial efficacy. In a study directly comparing hydrogel administration immediately, three days and one week post-MI, the three-day timepoint produced the greatest preservation of cardiac function and left ventricular geometry, with one-week delivery providing intermediate benefit, whereas immediate injection was ineffective [33]. Nonetheless, it is important to acknowledge that the effects of delivery timing are not universal. Hydrogel composition and bioactivity can markedly influence how the material interacts with the evolving infarct, with some studies reporting different optimal windows [34]. Finally, species-specific differences in infarct healing, ventricular geometry, and scar organization complicate direct comparison with the ovine model [35,36]. Altogether, ELR hydrogels may prove to be more effective when applied in models of moderate injury or during subacute phases of remodeling.

Despite the limited effects of CASCs or ELR hydrogel monotherapies, their combined delivery yielded the most consistent pattern of improvement across global and regional echocardiographic parameters. Although key outcomes such as LVEF and ventricular volumes did not reach statistical significance after correction for multiple testing, effect sizes were substantial and trends were reproducible across multiple outcome measurements, including AWT and WMSI. In the context of modest sample sizes inherent to invasive preclinical studies, effect sizes offer critical complementary information when *p*-values alone may underestimate biological relevance [37,38]. Similar patterns have been reported in other preclinical studies combining stem cells with injectable hydrogels, where combined strategies tend to outperform individual components across a range of cell types and biomaterial platforms. Notably, these benefits have been observed across distinct hydrogel formulations and cell types [39]. For instance, Ding et al. showed that combining MSCs with an injectable, ROS-scavenging and oxygen-generating hydrogel elicited a significantly greater improvement in LVEF compared with both MSC or hydrogel monotherapy [40]. Likewise, Mathieu et al. reported that MSCs delivered within a self-setting hydroxypropyl-methylcellulose hydrogel achieved superior preservation of function and scar architecture during both short- and mid-term follow-up compared with either strategy alone [41].

Analysis of regional wall motion further supported this interpretation. WMSI, derived qualitatively from segmental wall motion assessment, depends less on geometric assumptions than volumetric echocardiographic measures and is therefore less sensitive to deviations from normal ventricular geometry [42]. WMSI correlated strongly with ventricular volumes and anterior wall thickness and showed moderate correlations with LVEF, indicating that regional contractile impairment is a major contributor to global ventricular dysfunction in this model. The lower WMSI values and smaller estimated infarct extent observed in the combination group were consistent with the echocardiographic findings and reinforced the overall coherence of the dataset. WMSI is widely used clinically to quantify regional dysfunction and infarct extent and has been validated in rodent MI models as a reliable indicator of infarct severity [27,43]. These associations confirm internal consistency across modalities and reinforce the interpretation that the combination therapy produced concordant improvements in both regional mechanics and global functional performance. Nonetheless, WMSI has inherent limitations, as its subjective scoring makes it susceptible to variability related to image quality and observer interpretation [43]. Although WMSI is a semi-quantitative measure and subject to observer variability, the high interobserver agreement and its correlation with quantitative parameters observed in our study support its use in this context.

Transcriptomic profiling offered mechanistic insight into the molecular processes associated with the observed functional trends. The transcriptomic signature of the untreated MI group closely matched established molecular responses to MI and aligned with previously reported GSEA profiles from human acute MI, supporting the translational relevance of our model [44,45].

The combination therapy did not elicit a distinct global transcriptional response but rather reflected complementary contributions from each monotherapy. CASCs were associated with anti-inflammatory signatures, whereas the ELR hydrogel did not alter inflammatory pathways, consistent with the findings of Contessotto et al. [20]. While pathway-level analysis suggested attenuation of inflammatory signaling in the CASCs-treated groups (e.g., IL6–JAK–STAT3 signaling), targeted qPCR analysis indicated upregulation of individual inflammatory markers such as IL-6. This apparent discrepancy likely reflects the different analytical scales of both approaches. Pathway-level enrichment analysis integrates coordinated expression patterns across multiple genes, whereas the targeted qPCR panel reflects the regulation of a limited set of selected markers [46]. Consequently, differences between pathway-level and gene-level analyses may arise when individual genes do not fully reflect the overall transcriptional behavior of the pathway.

Beyond inflammation, CASCs uniquely depleted the Wnt/β-catenin and TGF-β signaling hallmarks. These pathways are widely implicated in maladaptive post-MI remodeling through their cooperative activation in cardiac fibroblasts, where they promote profibrotic gene expression and myofibroblast differentiation [47,48,49,50,51]. CASCs further reduced MYC targets and mTORC1 signaling, pathways operating downstream of Wnt/β-catenin and linked to fibrosis progression during chronic activation [52,53,54]. Collectively, these findings indicate that CASCs attenuate a Wnt–TGF-β–mTORC1 signaling axis, shifting the transcriptional landscape away from a profibrotic state. Consistent with this interpretation, all treatment groups showed depletion of the epithelial–mesenchymal transition (EMT) hallmark. The role of EMT in the adult heart has been described in MI, where activated epicardial cells can undergo EMT and contribute to the expansion of fibroblasts and post-infarct fibrosis [55,56]. Notably, endothelial–mesenchymal transition (EndMT), a subcategory of EMT, has been shown to provide a substantial portion of fibroblasts in a murine pressure overload model [57]. The downregulation of EMT-associated genes, together with reduced expression of Col1a2, α-SMA, and ATF3 across all therapies, as determined by qPCR, supports a shared antifibrotic effect.

All therapies also showed depletion of E2F target genes, which have been implicated in promoting myocardial injury under ischemic conditions [58]. In contrast, apoptosis and hypoxia hallmarks were selectively depleted in CASCs-containing groups, consistent with the close coupling between hypoxic stress, mitochondrial dysfunction, and apoptotic signaling after MI [59]. These findings suggest that CASCs more directly mitigate hypoxia- and apoptosis-related transcriptional programs, whereas the ELR hydrogel contributes more modestly through shared effects on cell cycle regulation. qPCR analyses corroborated these transcriptomic findings and further highlighted complementary effects on redox homeostasis. CASCs therapy upregulated NRF2 and GPx1 while downregulating NOX4, consistent with reduced oxidative stress. Together, these effects suggest convergence toward a more favorable oxidative environment, which is known to influence cardiomyocyte survival and ECM remodeling [60]. Metabolic remodeling further underscored this complementarity. The ELR hydrogel enriched the oxidative phosphorylation (OXPHOS) hallmark, suggesting partial preservation of mitochondrial metabolic function, consistent with previous observations using injectable myocardial matrix hydrogels [61,62]. In contrast, CASCs depleted the glycolysis hallmark. The combination therapy integrated these metabolic features, showing enrichment of OXPHOS together with depletion of glycolysis, consistent with a shift toward a more energetically favorable post-MI transcriptional profile.

The only gene set that was uniquely modulated by the combination therapy was Hedgehog signaling, which was depleted compared with MI. Hedgehog signaling is typically reactivated in ischemic injury, contributing to angiogenesis and cardioprotection in acute MI models [63,64]. However, sustained activation has been implicated in the promotion of fibrosis [65]. Given this dual role, its reduction could reflect either unintended suppression of repair pathways or beneficial antifibrotic signaling. Further investigation will be required to determine the functional significance of this unique transcriptional response.

Collectively, the findings of this proof-of-concept pilot study suggest that embedding CASCs within an ELR hydrogel may offer advantages over either monotherapy, as reflected by consistently favorable trends across functional, structural, and molecular endpoints. As a proof-of-concept pilot study, the present work was primarily designed to generate preliminary evidence and inform future investigations rather than to establish definitive therapeutic efficacy. Consequently, the study was limited by a small sample size and corresponding statistical uncertainty. Additional limitations include immediate post-MI treatment administration, which does not fully recapitulate the clinical scenario, particularly for autologous cell therapies, and the absence of direct measurements of in vivo cell retention. Furthermore, histological quantification of infarct size and fibrosis was not performed due to the limited tissue availability inherent to the pilot design, in which LV tissue was prioritized for molecular analysis. Although WMSI can be strongly associated with infarct size, histological validation of the observed effects remains an objective of future follow-up studies. Future studies should therefore evaluate delayed or subacute-stage delivery, ideally using minimally invasive approaches, while directly assessing in vivo cell retention and persistence over time. The in vitro characterization of CASCs within the ELR hydrogel as also limited to cell viability and spatial distribution, without quantitative assessment of proliferation or evaluation of other functional properties (paracrine activity, cardiomyogenic potential, …). Consequently, whether the ELR hydrogel modulates these properties remains to be established. Further characterization of CASCs properties within the ELR hydrogel will thus be important to better understand the interplay between these two therapeutic components.

## 4. Materials and Methods

### 4.1. Ethical Approval

All animal experimentation was conducted according to the EU Directive 2010/63/EU for animal experiments and was approved by the local ethics committee for animal experimentation (UHasselt, Diepenbeek, Belgium; ID 202234K, ID 202308). All animals were housed in a temperature-controlled environment (21 °C, 60% humidity), subjected to a natural day-night cycle of 12 h, and fed a standard pellet diet with access to water ad libitum. In total, 128 healthy female Sprague Dawley rats (Janvier Labs, Le Genest-Saint-Isle, France) of 8 weeks old were used.

### 4.2. ELRs Recombinant Synthesis and Functionalization

The two ELRs used in this work (HRGD6 and HE5), which have been previously described [20], were produced and supplied by Technical Proteins Nanobiotechnology, S.L. (TPNBT, S.L., Valladolid, Spain) as described elsewhere using recombinant DNA technology [66]. HRGD6 presents cell adhesion RGD sequences and HE5 includes MMP-sensitive domains for biodegradation (Supplementary Table S4). Both contain lysine residues as reactive sites, which make them suitable for subsequent chemical functionalization. Briefly, the corresponding synthetic genes were inserted into a pET-25b(+) expression vector (Novagen, Merck, Darmstadt, Germany) and subsequently introduced into the Escherichia coli BLR(DE3) strain (Novagen, Merck, Darmstadt, Germany). Positive clones were expanded in a controlled bioreactor system to promote high-yield ELR production. Protein purification was carried out by inverse transition cycling followed by dialysis, sterilized by filtration through a 0.22-μm membrane, and lyophilized. Subsequently, the ε-amine groups of lysine residues within the ELR sequences were chemically modified following established protocols [67]. HRGD6 was derivatized with azide moieties, achieving 55–65% functionalization, whereas HE5 was conjugated with cyclooctyne groups, achieving 30–45% modification. Functionalized polymers will be referred to as HRGD6-N_3_ and HE5-cc. In this way, the functionalized ELRs become suitable for subsequent covalent crosslinking via “click chemistry” for the formation of chemical hydrogels. Obtained polymers were characterized by hydrogen nuclear magnetic resonance (1H-NMR; NMR 500, Agilent Technologies, Santa Clara, CA, USA) and matrix-assisted laser desorption/ionization-time-of-flight (MALDI-TOF; Bruker Corporation, Billerica, MA, USA) to determine the functionalization degree and molecular weight (Supplementary Figure S4).

### 4.3. ELR Hydrogel Preparation and Characterization

HE5-cc and HRGD6-N_3_ were dissolved at 128.56 mg/mL and 35.71 mg/mL, respectively, and incubated on ice at 4 °C overnight to ensure complete dissolution. For hydrogel preparation at a final concentration of 50 mg/mL, the formulation consisted of 25% HE5-cyclooctyne and 50% HRGD6-azide, supplemented with either 25% phosphate-buffered saline (PBS; Thermo Fisher Scientific, Waltham, MA, USA) for acellular hydrogel characterization or an equivalent volume of cell suspension. Rheology experiments of the hydrogel were performed using a strain-controlled AR-2000ex rheometer coupled to a Peltier plate (TA Instruments, New Castle, DE, USA) to determine the viscoelasticity of the system. In this case, PBS was added to the hydrogel formulation instead of cell suspension, and hydrogels were prepared in 12 mm diameter Teflon molds. Experiments were performed on 12-mm parallel plates under oscillatory shear. Frequency sweeps were recorded between 0.01 and 10 Hz at 1% strain and 37 °C. Measurements were performed in triplicate.

### 4.4. Rat CASCs Isolation and Expansion

CASCs were isolated from the right atrial appendage (RAA) of 58 animals as described previously [29]. Rats received heparin (1000 units/kg, intraperitoneally (IP), LEO Pharma Nv, Lier, Belgium) and were sacrificed with sodium pentobarbital (200 mg/kg, IP, Dolethal, Vetoquinol, Lure, France). Following perfusion with PBS, hearts were harvested and the RAA was collected, minced into pieces of ~1 mm^3^, and enzymatically dissociated for 25 min at 37 °C in Hank’s Balanced Salt solution (HBSS) (Thermo Fisher Scientific) supplemented with collagenase NB4 (SERVA Electrophoresis GmbH, Heidelberg, Germany) and calcium chloride (CaCl_2_; VWR Chemicals, Leuven, Belgium). CASCs were isolated using the Aldefluor kit (STEMCELL Technologies, Vancouver, BC, Canada) and ALDH^+^ cells were flow-sorted (FACSAria II, BD Life Sciences, Franklin Lakes, NJ, USA), collected in TheraPEAK^®^ X-VIVO 15 medium (Lonza, Basel, Switzerland) supplemented with 20% FCS and 2% P/S, and seeded in 6-well plates. Cells were cultured at 37 °C and 5% CO_2_ with medium changes every 2–3 days. Rat CASCs were passaged at ~90% confluence, and passages 2–5 were used for intramyocardial injections.

### 4.5. CASCs-ELR Hydrogel Biocompatibility

CASCs from six rats were embedded in the ELR hydrogel at 10,000 cells per 150 µL gel and seeded in sterile µ-slide 8-well high glass bottom chambers (Ibidi, Gräfelfing, Germany). Each slide contained CASCs-hydrogel samples (*n* = 6), a hydrogel-only control, and a negative control in which cells in the ELR hydrogel were killed with Milli-Q water. One slide was used per timepoint (2, 3, 4, 6 and 16 days post-seeding). At each timepoint, samples were stained with the LIVE/DEAD™ Viability/Cytotoxicity Kit (Invitrogen, Waltham, MA, USA) with calcein-AM (2 µM) and ethidium homodimer-1 (EthD-1) (4 µM) for 35 min at room temperature, protected from light. Confocal imaging was performed on a Zeiss LSM 880 NLO AiryScan using an EC Plan-Neofluar 10×/0.30 objective lens and line-sequential 488 nm (calcein, Argon laser set to 60 µW before the objective) and 543 nm (EthD-1, HeNe laser set to 42 µW before the objective) excitation. Emission channels were set to detect 490–543 nm (calcein) and 644–745 nm (EthD-1) with a detector gain setting of 650 V for both detectors. Per sample, five fields of view (850 µm × 850 µm) were captured at standardized positions (center, left, right, top, and bottom). Z-stacks were acquired with a frame size of 512 × 512 pixels (voxel dimensions 1.66 µm × 1.66 µm × 10 µm) to a depth where no more cells could be visualized. The pixel dwell time was set to 4.10 µsec and no averaging was applied, for a total frame time of 1.26 s. The microscope incubator was maintained at 37 °C with humidified 5% CO_2_ in air. Image analysis was performed in Fiji/ImageJ (version 1.53t, NIH, USA). Live and dead cells were counted manually using maximum intensity projections created in ZEN Lite (v3.8), with verification across the z-stack. Calcein-positive (green) cells were counted as live, and EthD-1-positive (magenta) nuclei were counted as dead. Viability was calculated as the percentage of live cells out of the total number of live and dead cells. Data were reported descriptively as mean ± SD, as each timepoint consisted of independent biological replicates (i.e., distinct hydrogel constructs seeded with CASCs from different donor rats). Confocal z-stacks were imported into Zeiss Arivis Pro for volumetric visualization. Segmentation was performed using gap-filling, size filtering, and watershed separation, and each segmented object was pseudocolored to facilitate visualization of individual cells. Renderings were used qualitatively to assess spatial distribution of CASCs within the hydrogel.

### 4.6. Experimental Procedure

#### 4.6.1. Study Design

In total, 70 Female Sprague Dawley rats aged 8 weeks underwent the in vivo procedure. Of these, 15 (21%) died during surgery or within 24 h post-surgery, and 11 animals were excluded due to failed MI induction or unsuccessful injection. The remaining 44 animals were randomly assigned to one of five groups at the time of surgery by sequential assignment to the group with the lowest current sample size, maintaining balanced group sizes throughout the study. Thus, formal randomization with allocation concealment was not performed, and the surgeon was necessarily aware of the allocated treatment during the procedure. All subsequent analyses (echocardiography and WMSI, invasive hemodynamics, RNA-seq, qPCR) were performed by 2 independent investigators blinded to group allocation, using anonymized samples identified solely by animal number. The groups included in this study were: SHAM (*n* = 7), MI (*n* = 9), MI with ELR hydrogel injection (MI+H, *n* = 9), MI with CASCs transplantation (MI+C, *n* = 10), and MI with CASCs–ELR hydrogel injection (MI+C+H, *n* = 9). SHAM animals underwent the surgery without left anterior descending coronary artery (LAD) ligation.

**Figure 6:**
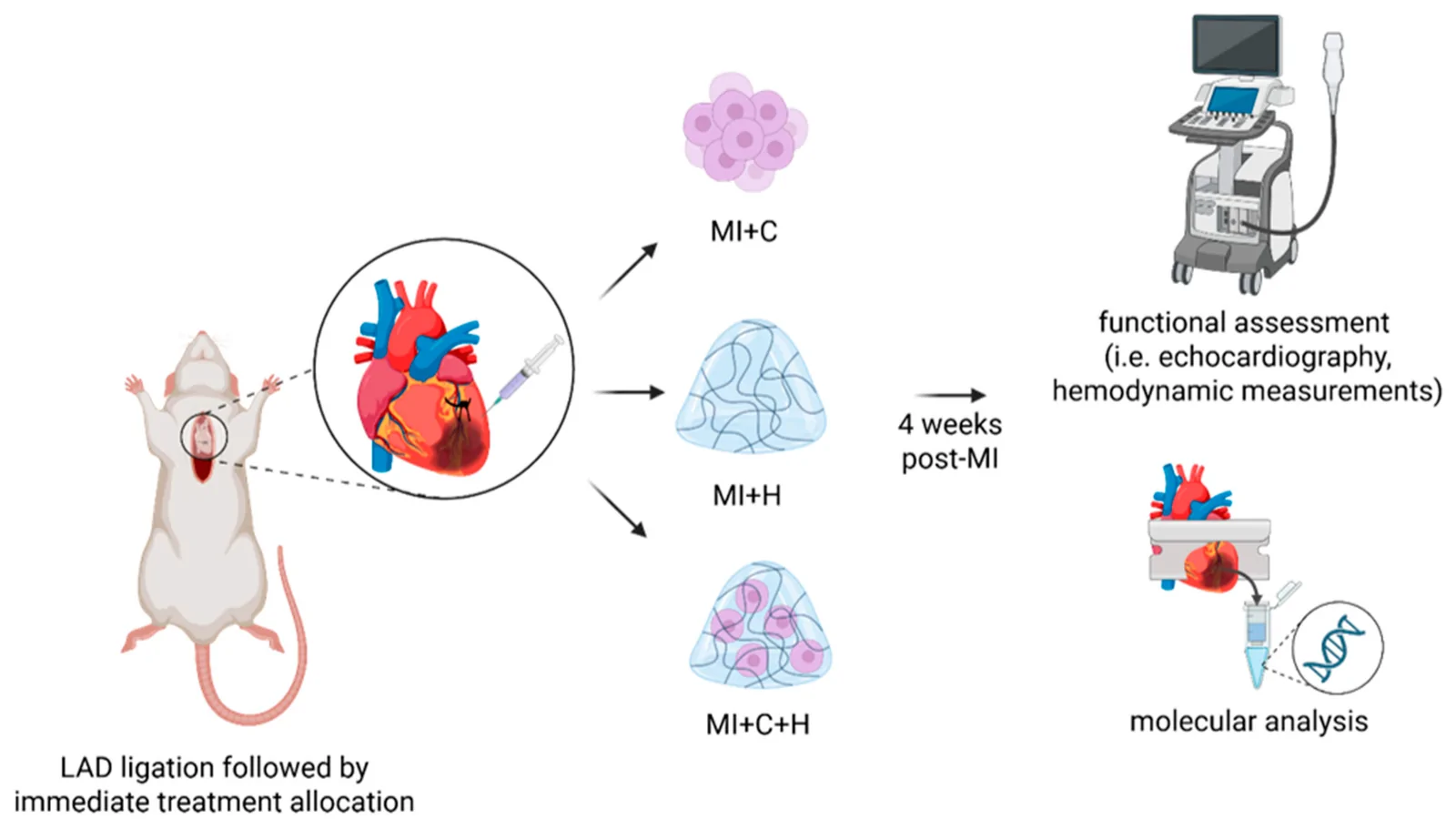
Graphical overview of the study design. On day 0, MI was induced by permanent LAD ligation, immediately followed by intramyocardial injection of the assigned treatment into the peri-infarct zone. MI animals received no intramyocardial injection. SHAM animals underwent the surgical procedure without LAD ligation and intramyocardial injection. Four weeks after surgery, cardiac function was assessed by echocardiography and invasive hemodynamic measurements. Subsequently, animals were sacrificed and the left ventricle (LV) was harvested for molecular analyses. This figure was created with BioRender.com.

The experimental timeline was as follows (Figure 6): on day 0, MI was induced by permanent LAD ligation, immediately followed by intramyocardial injection of the assigned treatment into the peri-infarct zone. Four weeks after surgery, cardiac function was assessed by echocardiography and invasive hemodynamic measurements. Subsequently, animals were sacrificed and the left ventricle (LV) was harvested for molecular analyses.

The primary outcomes were cardiac function (echocardiography and hemodynamics) at 4 weeks post-MI. Secondary outcomes were the molecular responses of the LV tissue, as described below. The complete study design, including group allocation, treatment administration, and outcome assessment, is depicted graphically in Figure 6.

#### 4.6.2. Surgical Procedure and Treatment Administration

Permanent LAD ligation was performed as described before [68]. Briefly, rats were anesthetized with 5% isoflurane supplemented with oxygen (2 L/min). Following orotracheal intubation, rats were ventilated at 55 breaths per minute with a tidal volume of 2 mL. Anesthesia was reduced to 2.5% isoflurane and a prophylactic dose of buprenorphine was administered intramuscularly (0.02 mg/kg, intramuscular (IM); Richter Pharma AG, Wels, Austria). A left thoracotomy was performed in the third intercostal space. The LAD was permanently ligated using an 8-0 Prolene suture (Ethicon Inc., Raritan, NJ, USA). MI was confirmed by immediate pallor of the left ventricle (LV) distal to the ligation. After successful MI, 80 µL of the assigned treatment was injected intramyocardially into the peri-infarct zone using a 30 G needle. For the cell-based treatments, 2 × 10^6^ CASCs were either suspended in ELR hydrogel (50 mg/mL) in the MI+C+H group or incorporated into a Matrigel-based construct containing 48.3% cells suspended in X-VIVO medium, 16% Matrigel (Corning, New York, NY, USA), 34% rat tail collagen (Corning, New York, NY, USA), and 1.7% NaHCO_3_ (VWR Chemicals, Leuven, Belgium) in the MI+C group to prevent immediate washout after intramyocardial injection. The anesthesia was reduced to 1% isoflurane after successful delivery. Both the intercostal space and dermal incision were closed using a 5-0 Vicryl Rapide suture (Ethicon). Following signs of awakening, the animals were extubated and were subjected to post-operative solitary housing for 24 h. An additional dose of buprenorphine was administered 24 h post-surgery for pain management. Four weeks later, end-point echocardiography and invasive hemodynamic measurements were performed (as described below). For sacrifice, animals were injected with heparin (1000 units/kg, IP) and sodium pentobarbital (200 mg/kg, IP). Following perfusion with Tyrode solution (137 mM NaCl, 5.4 mM KCl, 0.5 mM MgCl_2_, 1 mM CaCl_2_, 11.8 mM HEPES, 10 mM D-glucose), hearts were harvested, weighed, and the LV isolated. For downstream analysis, LV tissue was snap-frozen in liquid nitrogen, powdered, and stored at −80 °C.

### 4.7. Echocardiography

Using the Vevo 3100 system and 21 MHz MX250 linear probe (FUJIFILM VisualSonics Inc., Toronto, ON, Canada), transthoracic echocardiography was performed at baseline and four weeks after MI, as described previously [29]. Briefly, rats were anesthetized with 2% isoflurane supplemented with oxygen (1 L/min). The fur was shaved and depilatory cream was applied to remove interfering artefacts. Heart rate, respiratory rate, and electrocardiogram (ECG) were monitored throughout imaging. Parasternal long axis (PSLAX) and short axis (SAX) views at mid-ventricular level (papillary muscle) were obtained in brightness mode (B-mode) and motion mode (M-mode) to evaluate cardiac function, wall motion, and infarct size, using the Vevo LAB software (v5.11.0). Additionally, 4D images were acquired to generate SAX ECG-gated kilohertz visualization (EKV) images at basal, mid-ventricular, and apical levels, which were used for regional LV wall motion scoring (16-segment model). The wall motion score index (WMSI) was calculated as the sum of all segment scores divided by 16, and infarct size (%) was determined as the number of segments with a score > 2 divided by 16, multiplied by 100.

### 4.8. Invasive Hemodynamic Measurements

Invasive hemodynamic measurements were performed right before sacrifice, as described before [29]. In short, animals were anesthetized with 2% isoflurane supplemented with oxygen (2 L/min). An SPR-320 Mikro-Tip single pressure catheter (Millar Inc., Pearland, TX, USA), connected to a quad-bridge amplifier and PowerLab26T module (AD Instruments, Dunedin, New Zealand) to transfer the pressure data to LabChart v7.3.7 software (AD Instruments), was guided to the LV via the right carotid artery. Immediately before measurement, the catheter was calibrated according to the guidelines of AD Instruments. Here, the catheter was soaked in 37 °C isotonic saline for 30 min prior to balancing and calibrating. Functional parameters were subsequently obtained from the LV pressure traces.

### 4.9. RNA Sequencing and qPCR

Total mRNA derived from LV tissue was isolated using the RNeasy Fibrous Tissue kit (Qiagen, Venlo, The Netherlands) following the manufacturer’s instructions. RNA yield and purity were assessed with a NanoDrop 2000 spectrophotometer (Isogen Life Science, Utrecht, The Netherlands). Samples were submitted to Novogene Bioinformatics Technology Co., Ltd. (Beijing, China) for bulk RNA sequencing. Messenger RNA was purified using poly-T oligo-attached magnetic beads, and first- and second-strand cDNA was synthesized with random hexamer primers. Non-stranded cDNA libraries were quantified with Qubit fluorometry and sequenced on an Illumina NovaSeq X Plus platform (paired-end 150 bp reads). Raw FASTQ files were processed using the nf-core/rnaseq pipeline (v3.14.0) via the Flemish Supercomputer Center (VSC) Linux environment with Nextflow configuration. Sample quality was assessed via MultiQC reports, and low-quality samples were excluded (Supplementary Table S5). A PCA plot was created to examine clustering of the data (Supplementary Figure S5). Gene counts were used for differential expression analysis in R (DESeq2, v4.2.2), with adjusted *p*-values < 0.05 considered significant. Here, the design formula compared each treatment with MI separately. Gene set enrichment analysis was performed using MSigDBR and fgsea, and significant hallmark gene sets were visualized. To examine expression levels of a predefined set of MI-related genes, quantitative polymerase chain reaction (qPCR) was performed. First, mRNA (1000 ng) was reverse-transcribed with qScript cDNA SuperMix (Quantabio, Beverly, MA, USA) to obtain a cDNA concentration of 5 ng/µL. qPCR was conducted on cDNA samples (12.5 ng per reaction) using the QuantStudio 3 Real-Time PCR System (Thermo Fisher Scientific) and was based on SYBR Green (Thermo Fisher Scientific) as a reagent. Primers (Supplementary Table S6) were designed in the mRNA coding sequence and diluted based on primer efficiency analysis. Reference genes were selected using GeNorm, and data were analyzed with the ΔΔCt method following MIQE guidelines [69,70]. Relative gene expression values, normalized to the most stable housekeeping genes (YWHAZ, RPL13A, HPRT), were visualized using a heatmap generated in R (version 2023.12.1+402) with the ComplexHeatmap package. For each gene, normalized expression values were averaged per experimental group and Z-score normalized to facilitate comparison of relative expression patterns across conditions. Hierarchical clustering was applied to experimental groups to reveal biologically meaningful similarities, while genes were organized according to predefined functional categories.

### 4.10. Statistical Analysis

Statistical analyses were performed in Graphpad Prism (version 10.5.0). Given the small sample sizes (*n* ≤ 10), non-parametric statistical methods were used throughout because the assumption of normality could not be reliably evaluated. Comparisons between two groups were performed using the Mann–Whitney U test. For analyses involving more than two groups, pairwise Mann–Whitney tests were conducted for all group combinations, and *p*-values were adjusted for multiple testing using the Holm–Bonferroni method. When appropriate, overall group differences were additionally evaluated using the Kruskal–Wallis test. As this study was designed as a proof-of-concept pilot study, effect sizes were reported as mean differences with corresponding 95% confidence intervals and standardized effect sizes (Hedges’ g) to help interpretation of the magnitude and precision of observed effects. Interobserver agreement for LVEF and WMSI was quantified using Bland–Altman analysis, reporting mean bias and 95% limits of agreement. Correlations between regional and global echocardiographic measures were assessed using Spearman’s rank correlation coefficient. Linear regression lines were included for visualization only. Data are displayed as Tukey-style box-and-whisker plots (median, IQR, whiskers = 1.5 × IQR), with individual outliers beyond the whisker range plotted as separate points. Summary descriptive statistics (*n*, mean ± SD, median [IQR]) are reported in Supplementary Tables S2 and S3. Statistical significance was defined as Holm–Bonferroni-adjusted *p* < 0.05; effect sizes and confidence intervals were additionally considered when interpreting the exploratory findings.

## 5. Conclusions

This proof-of-concept pilot study demonstrates the feasibility of ELR hydrogel-mediated CASC delivery in a rat model of MI and provides initial evidence that the combination therapy may be advantageous over either monotherapy, with consistently favorable trends across functional, structural, and molecular endpoints. The transcriptional profiles observed here suggest that the two components act through distinct mechanisms rather than a shared synergistic program. While these findings require confirmation in adequately powered studies, they support further investigation of ELR hydrogel-mediated CASCs delivery as a potential approach for enhancing cardiac repair after MI.

## Figures and Tables

**Table 1:** Hemodynamic parameters 4 weeks post-surgery.

Parameter	SHAM (*n* = 7)	MI (*n* = 9)	MI+C (*n* = 6)	MI+H (*n* = 9)	MI+C+H (*n* = 7)
LVSP (mmHg)	91 [85;96]	88 [85;101]	94 [86;103]	87 [83;100]	92 [82;95]
LVEDP (mmHg)	4.591 [4.466;6.202]	5.620 [3.422;13.040]	4.070 [2.605;10.270]	4.269 [2.143;5.950]	3.609 [3.192;6.485]
dP/dt_max_ (mmHg/s)	6595 [5349;7913]	6315 [5403;8148]	6783 [5527;7805]	5942 [5677;7416]	6385 [5636;7842]
dP/dt_min_ (mmHg/s)	−6680 [−7844;−6200]	−5933 [−6975;−5041]	−6519 [−6885;−5386]	−5678 [−7668;−5050]	−6258 [−7283;−4457]
Tau (s)	0.0115 [0.0106;0.0121]	0.0135 [0.0116;0.0165]	0.0139 [0.0125;0.0164]	0.0137 [0.0115;0.0143]	0.0147 [0.0134;0.0176]
Heart rate (bpm)	342.7 [339.1;365.4]	365.9 [333.3;422.3]	358.6 [326.1;371.4]	366.4 [325.3;401.5]	322.5 [300.7;368.0]

LVSP, left ventricular systolic pressure; dP/dt_max_, maximum rate of pressure rise; dP/dt_min_, maximum rate of pressure fall; Tau, time constant of LV relaxation; LVEDP, left ventricular end-diastolic pressure. Values are presented as median and interquartile range (IQR). No significant differences were observed between groups (Mann–Whitney test with Holm–Bonferroni correction).

## Data Availability

The data presented in this study are available on request from the corresponding author due to applicable confidentiality and intellectual property considerations.

## Supplementary Materials

The following supporting information can be downloaded at https://www.mdpi.com/article/10.3390/ijms27219563/s1.

## Abbreviations

The following abbreviations are used in this manuscript:

1H-NMR	Hydrogen nuclear magnetic resonance
α-SMA	α-Smooth muscle actin
ALDH	Aldehyde dehydrogenase
Akt	Protein kinase B
ATF3	Activating Transcription Factor 3
AW	Anterior wall
AWTd	Anterior wall thickness in diastole
AWTs	Anterior wall thickness in systole
BM-MNCs	Bone marrow-derived mononuclear cells
CASCs	Cardiac atrial appendage stem cells
cDNA	Complementary DNA
Col1a2	Collagen type 1 alpha 2
CPCs	Cardiac progenitor cells
DNA	Deoxyribonucleic acid
dP/dt_max_	Maximum rate of pressure rise
dP/dt_min_	Minimum rate of pressure rise
E2F	E2 promoter binding factor
ECG	Electrocardiogram
ECM	Extracellular matrix
EDV	End-diastolic volume
EKV	Electrocardiogram-gated kilohertz visualization
ELR	Elastin-like recombinamer
EMT	Epithelial–mesenchymal transition
endMT	Endothelial–mesenchymal transition
ESV	End-systolic volume
EthD	Ethidium homodimer
FCS	Fetal calf serum
GPx1	Glutathione peroxidase 1
GSEA	Gene set enrichment analysis
HBSS	Hank’s Balanced Salt Solution
HF	Heart failure
HPRT	Hypoxanthine-fosforibosyltransferase
IHD	Ischemic heart disease
IL6	Interleukin-6
IP	Intraperitoneal
iPSCs	Induced pluripotent stem cells
IQR	Interquartile range
JAK	Janus kinase
LAD	Left anterior descending coronary artery
LV	Left ventricle
LVEDP	Left ventricular end-diastolic pressure
LVEF	Left ventricular ejection fraction
LVSP	Left ventricular systolic pressure
MALDI-TOF	Matrix-assisted laser desorption/ionization time-of-flight
MI	Myocardial infarction
MMP	Matrix metalloproteinase
MSCs	Mesenchymal stem cells
mTORC1	Mechanistic target of rapamycin complex 1
NES	Normalized enrichment score
NMR	Nuclear magnetic resonance
NOX4	NADPH oxidase 4
NRF2	Nuclear factor erythroid 2-related factor 2
OXPHOS	Oxidative phosphorylation
P/S	Penicillin/streptomycin
PBS	Phosphate-buffered saline
PI3K	Phosphoinositide 3-kinase
PSLAX	Parasternal long axis
QC	Quality control
qPCR	Quantitative polymerase chain reaction
RAA	Right atrial appendage
RNA	Ribonucleic acid
ROS	Reactive oxygen species
RPL13A	Ribosomal Protein L13a
SAX	Short axis
SD	Standard deviation
STAT3	Signal transducer and activator of transcription 3
Tau	Time constant for isovolumetric relaxation
TGF-β	Transforming growth factor β
VSC	Flemish Supercomputer Center
WMSI	Wall motion score index
YWHAZ	Tyrosine 3-monooxygenase/tryptophan 5-monooxygenase activation protein zeta
