# Supplementary material for: Cardiac Atrial Appendage Stem Cells Embedded in Elastin-like Recombinamer Hydrogel for Cardiac Repair After Acute MI: A Pilot Study
Source: Int J Mol Sci. 2026 Oct 26;27(21):9563. doi: 10.3390/ijms27219563 (PMC13762472; doi:10.3390/ijms27219563)
Supplement: Supplementary file 1 [file ijms-27-09563-s001.zip › ijms-4613489-supplementary.pdf]

Table S1. Prospective sample size estimation for future confirmatory studies.

Parameter	Value
Primary endpoint	Left ventricular ejection fraction
Comparison	MI vs MI+C+H
Effect size (Cohen's d)	0.86
Statistical test	Two-sided independent-samples t-test
α	0.05
Desired power	80%
Allocation ratio	1:1
Required sample size	22 animals per group

Sample size estimation was performed in G*Power 3.1.9.7 using Cohen's d, as required by the software. Hedges' g is reported in the main results because it corrects for small-sample bias. The estimate is intended to guide future confirmatory studies and should be interpreted cautiously given the uncertainty of effect-size estimates from pilot studies.

Table S2. Descriptive statistics for quantitative echocardiographic parameters.

Parameter		Sample size (n)	Mean	Standard deviation (SD)	Median	Interquartile range
LVEF (%)	SHAM	7	64,51	4,969	64,69	61,01 - 67,79
	MI	9	44,31	12,38	49,09	30,50 - 52,72
	MI+C	10	51,01	13,98	47,95	41,12 - 63,17
	MI+H	9	49,82	18,60	45,26	35,82 - 70,33
	MI+C+H	9	55,47	12,97	54,83	45,91 - 66,94
EDV (μL)	SHAM	7	409,49	73,91	446,54	335,08 - 473,31
	MI	9	522,81	84,69	542,08	455,81 - 590,99
	MI+C	10	487,08	97,07	517,31	388,31 - 546,53
	MI+H	9	454,15	79,38	498,97	359,25 - 518,15
	MI+C+H	9	425,52	69,26	401,13	375,56 - 499,89
ESV (μL)	SHAM	7	144,67	31,25	150,43	122,50 - 155,09
	MI	9	296,73	99,46	295,91	206,63 - 393,79
	MI+C	10	243,77	93,92	266,83	159,22 - 320,53
	MI+H	9	232,41	106,3	238,43	127,51 - 325,25
	MI+C+H	9	195,14	81,56	188,72	128,59 - 256,68
AWT;s (mm)	SHAM	7	2,89	0,37	2,92	2,69 - 3,19
	MI	9	1,36	0,65	1,33	0,79 - 2,02
	MI+C	10	1,25	0,83	0,99	0,57 - 1,87
	MI+H	9	0,95	0,35	0,97	0,67 - 1,23
	MI+C+H	7	1,96	0,80	1,87	1,35 - 2,48
AWT;d (mm)	SHAM	7	1,71	0,26	1,65	1,46 - 2,04
	MI	9	1,03	0,29	1,164	0,74 - 1,31
	MI+C	10	0,98	0,32	0,9552	0,73 - 1,16
	MI+H	9	0,74	0,23	0,7335	0,57 - 0,87
	MI+C+H	7	1,47	0,60	1,296	0,93 - 1,85
AW thickening (%)	SHAM	7	71,26	27,00	58,84	54,90 - 101,70
	MI	9	26,02	29,48	16,72	-1,90- 53,62
	MI+C	10	20,54	50,08	17,97	-19,12 - 35,36
	MI+H	9	29,45	34,41	38,27	-7,08 - 46,75
	MI+C+H	7	27,67	15,01	33,23	13,23 - 43,89

LVEF, left ventricular ejection fraction, EDV, end-diastolic volume; ESV, end-systolic volume; AWT;s, anterior wall thickness in systole; AWT;d, anterior wall thickness in diastole; AW thickening, anterior wall thickening.

Table S3. Descriptive statistics for wall motion score derived parameters.

Parameter		Sample size (n)	Mean	Standard deviation (SD)	Median	Interquartile range
WMSI	SHAM	7	1,05	0,059	1,00	1,00 - 1,13
	MI	7	1,68	0,296	1,69	1,44 - 1,94
	MI+C	9	1,63	0,240	1,63	1,41 - 1,75
	MI+H	9	1,70	0,279	1,75	1,50 - 1,94
	MI+C+H	8	1,44	0,236	1,41	1,22 - 1,63
Infarct (%)	SHAM	7	2,714	5,057	0,00	0,00 - 6,00
	MI	7	34,14	11,28	38,00	31,00 - 38,00
	MI+C	9	32,78	7,645	31,00	28,00 - 38,00
	MI+H	9	31,33	12,27	31,00	25,00 - 41,00
	MI+C+H	8	23,63	9,149	22,00	14,50 - 31,00

WMSI, wall motion score index.

Table S4. Amino acid sequence of the ELRs.

ELR	Sequence
HRGD6	MGSSHHHHHHSSGLVPRGSHMESLLP $[(VPGIG)_2(VPGKG)(VPGIG)_2]_2$<br>AVTGRGDSPASS $[(VPGIG)_2$ VPGKG $(VPGIG)_2]_2)_6$ V
HE5	MGSSHHHHHHHHGLVPRGSHMG-KKK-$[(VPGVG)_2VPGEG(VPGVG)_2]_5$-GGGGG<br>PMGPSGPW GGGGGVGGGG QPQGLAK GGGGGVGGGGG PQGIWGQ GGGG-<br>$[(VPGVG)_2VPGEG(VPGVG)_2]_5$-V GGGGGKKKGGGGG-<br>$[(VPGVG)_2VPGEG(VPGVG)_2]_5$-GGGGG PMGPSGPW<br>GGGGGVGGGGQPQGLAKGGGGGV GGGGGPQGIWGQGGGG-<br>$[(VPGVG)_2VPGEG(VPGVG)_2]_5$-KKK

Table S5. QC information of the RNAseq analysis.

Group	Sample	RIN	Sample QC	M total sequences	% aligned	% duplication	%rRNA	Final analysis
SHAM	EH3	3.2	fail (unqualified RIN)	/	/	/	/	/
	EH4	8.9	pass	55.0	89.5%	54.3%	0.02%	included
	EH9	9.6	pass	48.8	89.1%	52.8%	0.01%	included
	EH10	9.7	pass	67.8	87.9%	53.2%	0.01%	included
	EH44	9.5	pass (back-up for EH3)	42.7	90.1%	53.3%	0.01%	included
MI	EH1	3.1	fail (unqualified RIN)	/	/	/	/	/
	EH2	9.3	pass	56.1	89.7%	51.8%	0.02%	included
	EH5	9.6	pass	54.4	91.8%	53.1%	0.01%	included
	EH17	9.9	pass	42.4	90.4%	49.2%	0.01%	included
	EH43	4.6	pass (back-up for EH1)	59.5	83.2%	84.4%	0.00%	excluded
MI+C	EH12	9.9	pass	45.3	88.5%	47.1%	0.00%	included
	EH13	9.8	pass	44.3	84.8%	92.1%	0.02%	excluded
	EH18	9.9	pass	45.2	91.1%	49.0%	0.01%	included
	EH19	9.9	pass	44.5	90.9%	46.0%	0.01%	included
MI+H	EH6	9	pass	56.5	87.7%	50.0%	0.02%	included
	EH7	9.5	pass	50.4	91.2%	51.3%	0.04%	included
	EH8	8.4	pass	46.3	87.0%	56.4%	0.01%	included
	EH16	9.9	pass	54.0	90.6%	48.2%	0.01%	included
MI+C+H	EH11	9.6	pass	43.0	91.0%	50.7%	0.01%	included
	EH14	9.9	pass	44.4	91.3%	46.4%	0.00%	included
	EH15	9.9	pass	42.2	92.1%	46.6%	0.00%	included
	EH20	9.9	pass	47.0	91.0%	46.2%	0.00%	included

Samples were sequenced by Novogene Bioinformatics Technology Co., Ltd. (Beijing, China). Here, RNA integrity (RIN) was checked and sample quality control was performed by the company. Briefly, sample EH1 and EH3 failed RIN check and were not sequenced. Instead, samples EH43 and EH44 served as respective back-ups. After running the nf-core/rnaseq pipeline (v3.14.0), an additional quality control was performed via MultiQC reports. These reports included sequencing depth per sample (M total sequences), mapping rate to the reference genome (% aligned), library complexity (% duplication), and contamination (%rRNA). Based on this quality control, EH13 and EH43 were left out of the downstream analysis, which included a total of 18 samples. Sample sizes per group were; SHAM: n = 4, MI: n = 3, MI+C: n= 3, MI+H: n = 4, MI+C+H: n = 4.

Table S6. qPCR primer sequences.

Genes	Forward primer	Reverse primer
YWHAZ	5′ - GAT GAA GCC ATT GCT GAA CTT G - 3′	5′ - GTC TCC TTG GGT ATC CGA TGT C - 3′
RPL13A	5′ - GGA TCC CTC CAC CCT ATG ACA - 3′	5′ - CTG GTA CTT CCA CCC GAC CTC - 3′
HPRT	5′ - TCC CAG CGT GAT TAG TG - 3′	5′ - GCA AGT CTT TCA GTC CTG TCC - 3′
TGF-β3	5′ - ACC GCA ACA ACG CAA TCT ATG - 3′	5′ - GCA CTG CTT CCC GAA TGT CT - 3′
IL-6	5′ - TAG TCC TTC CTA CCC CAA CTT CC - 3′	5′ - TTG GTC CTT AGC CAC TCC TTC - 3′
Itgβ1	5′ - AAA ATG GAC GAA AGT GCT CTA AC - 3′	5′ - TGG GAC TTG CTG GGA TGC - 3′
ATF3	5′ - ATG TCA GTC ACC AAG TCT GAG G - 3′	5′ - TTC GGC ATT CAC ACT CTC CA - 3′
TNF-α	5′ - GTT GCC TCC CCC TTT TCT TT - 3′	5′ - CCT GGT CAC CAA ATC AGC ATT - 3′
CD68	5′ - CAC TTG GCT CTC TCA TTC CCT - 3′	5′ - GGT GAG AAT GTC CAC TGT GCT - 3′
IL-1β	5′ - CAA AAA TGC CTG GTG CTG TCT - 3′	5′ - GCT CAT GGA GAA TAC CAC TTG TTG - 3′
GPx1	5′ - CAA TCA GTT CGG ACA TCA GGA GAA - 3′	5′ - CTC ACC ATT CAC CTC GCA CTT - 3′
SOD2	5′ - AGC TGC ACC ACA GCA AGC AC - 3′	5′ - TCC ACC ACC CTT AGG GCT CA - 3′
NRF2	5′ - CAG CTA CTC CCA GGT TGC CC - 3′	5′ - ATA GCT CCT GCC AAA CTT GCT - 3′
NOX4	5′ - TCA TGG ATC TTT GCC TGG AGG GTT - 3′	5′ - AGG TCT GTG GGA AAT GAG CTT GGA - 3′
COL3A1	5′ - AAC TGG AGC ACG AGG TCT TG - 3′	5′ - CGT TCC CCA TTA TGG CCA CT - 3′
SOD1	5′ - GCT TCT GTC GTC TCC TTG CT - 3′	5′ - CAC AAC TGG TTC ACC GCT TG - 3′
NFκB1	5′ - GAC AAC TAT GAG GTC TCT GGG G - 3′	5′ - CTG GAT CAC TTC AAT GGC CTC - 3′
COL1A2	5′ - GAA GAC CCC TTT CTC CTG GC - 3′	5′ - AGG GTG TTC AAG GTG GCA AA - 3′
α-SMA	5′ - GAG CGT GGC TAT TCC TTC GTG - 3′	5′ - CAG TGG CCA TCT CAT TTT CAA AGT - 3′
CCL2	5′ - CAG GTG TCC CAA AGA AGC TGT A - 3′	5′ - CTG AAG TCC TTA GGG TTG ATG C - 3′
TIMP2	5′ - GGA CAC GCT TAG GAT CAC CCA GA - 3′	5′ - GTC CAT CCA GAG GCA CTC ATC C - 3′
LOX3	5′ - AGC TGC CAC CAA CAT TAC CA - 3′	5′ - GGG ACT CAA CCC CTG TGT G - 3′
MMP2	5′ - AGA CAA AGA GTT GGC AGT GCA AT - 3′	5′ - CTG TAT GTG ATC TGG TTC TTG TCC C - 3′

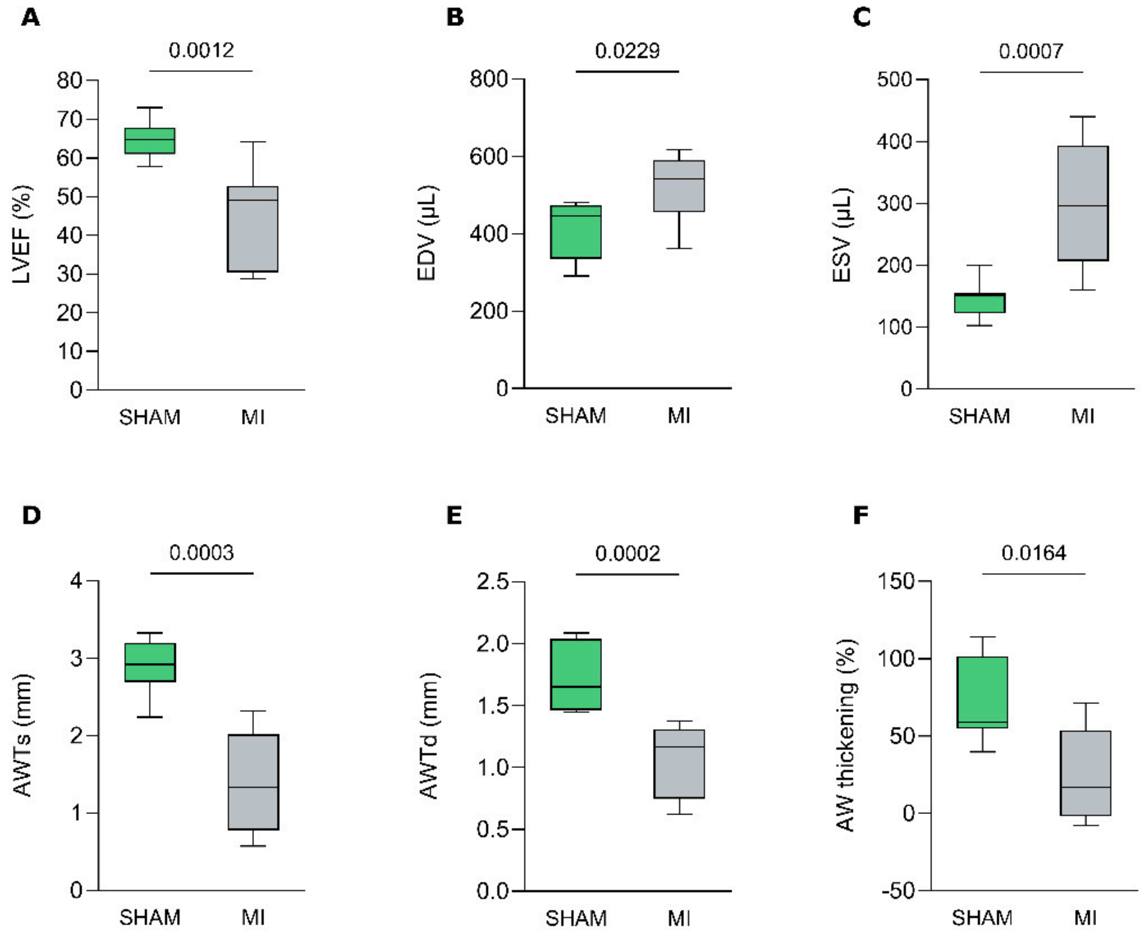

Supplementary figure S1. Model validation SHAM vs MI. Echocardiographic assessment of global LV performance four weeks after surgery. (**A**) Ejection fraction (EF), (**B**) end-diastolic volume (EDV), (**C**) end-systolic volumes (ESV), (**D**) anterior wall thickness in systole (AWT;s), (**E**) anterior wall thickness in diastole (AWT;d), (**F**) anterior wall thickening. Data are presented as Tukey-style box-and-whisker plots, showing the median, interquartile range (IQR, 25th–75th percentile), and whiskers extending to 1.5 × IQR. Exact p-values from Mann–Whitney tests are displayed.

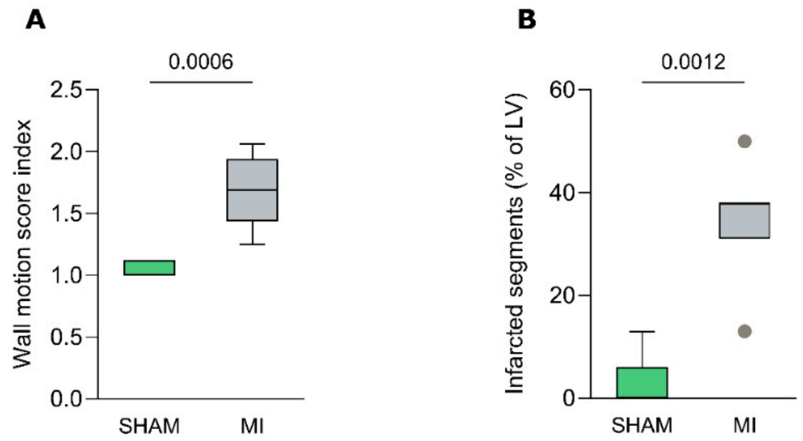

Supplementary figure S2. WMSI model validation SHAM vs MI. Regional dysfunction quantified from wall motion scoring. (**A**) Wall motion score index (WMSI), (**B**) Infarct size, expressed as the percentage of dysfunctional LV segments. Data are presented as Tukey-style box-and-whisker plots (median, IQR, whiskers = 1.5×IQR). Exact p-values from Mann–Whitney tests are displayed within each panel.

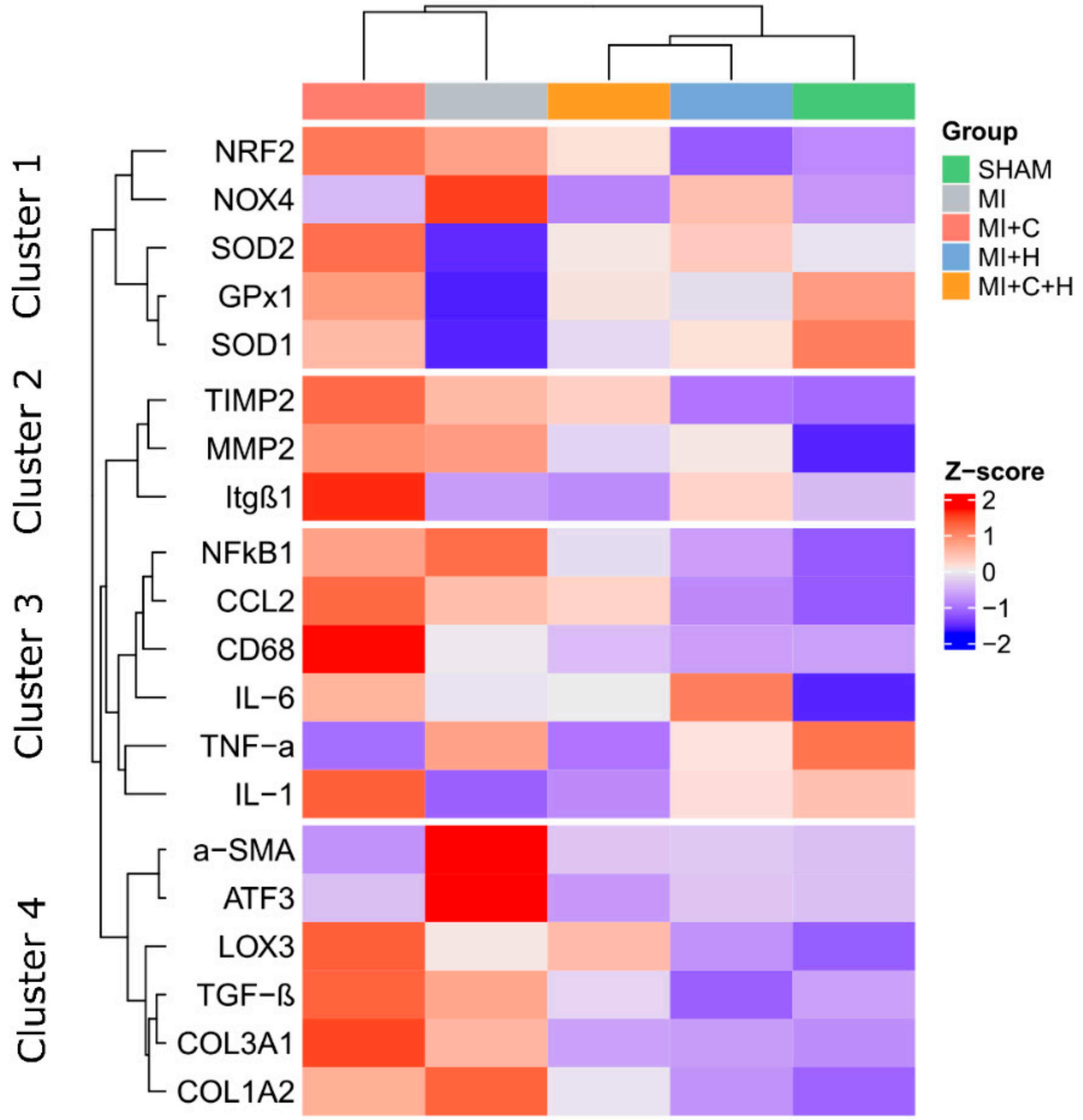

Supplementary figure S3. Gene Expression Validation Across Therapies by qPCR. Heatmap of selected genes quantified by qPCR, showing relative expression levels across groups after normalization to housekeeping genes. Genes are grouped into functional categories; Cluster 1: oxidative stress and antioxidant defense (NRF2, NOX4, SOD1, SOD2, GPx1), Cluster 2: extracellular matrix remodeling (TIMP2, MMP2, Itgβ1), Cluster 3: inflammatory signaling (NFκB1, CCL2, CD68, IL-6, TNF-α, IL-1), and Cluster 4: fibrotic remodeling (α-SMA, ATF3, LOX3, TGF-β, COL3A1, COL1A2).

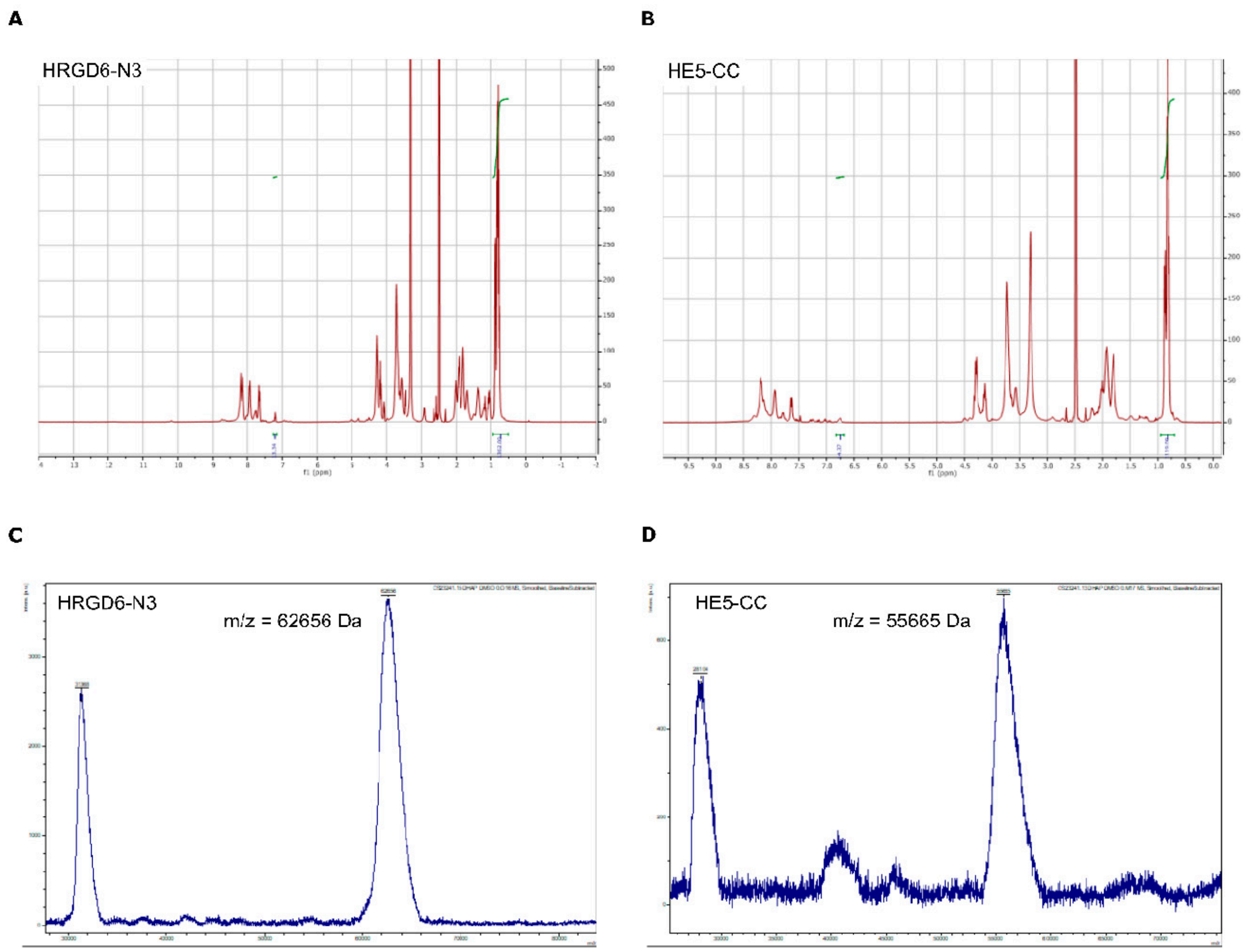

Supplementary figure S4. NMR and MALDI-TOF characterization of HE5-cc and HRGD6-N_3. (**A**) 1H-NMR spectra of HRGD6-N3 and HE5-cc in DMSO-d6 indicating a modification degree of ~58 % (13-14 modified lysines out of 24) for HRGD6-N3, and ~44% (4-5 modified lysines out of 9) for HE5-cc. (**B**) MALDI-TOF mass spectra of HRGD6-N3 and HE5-cc showing molecular weights of ~62656Da and ~55665Da, respectively.

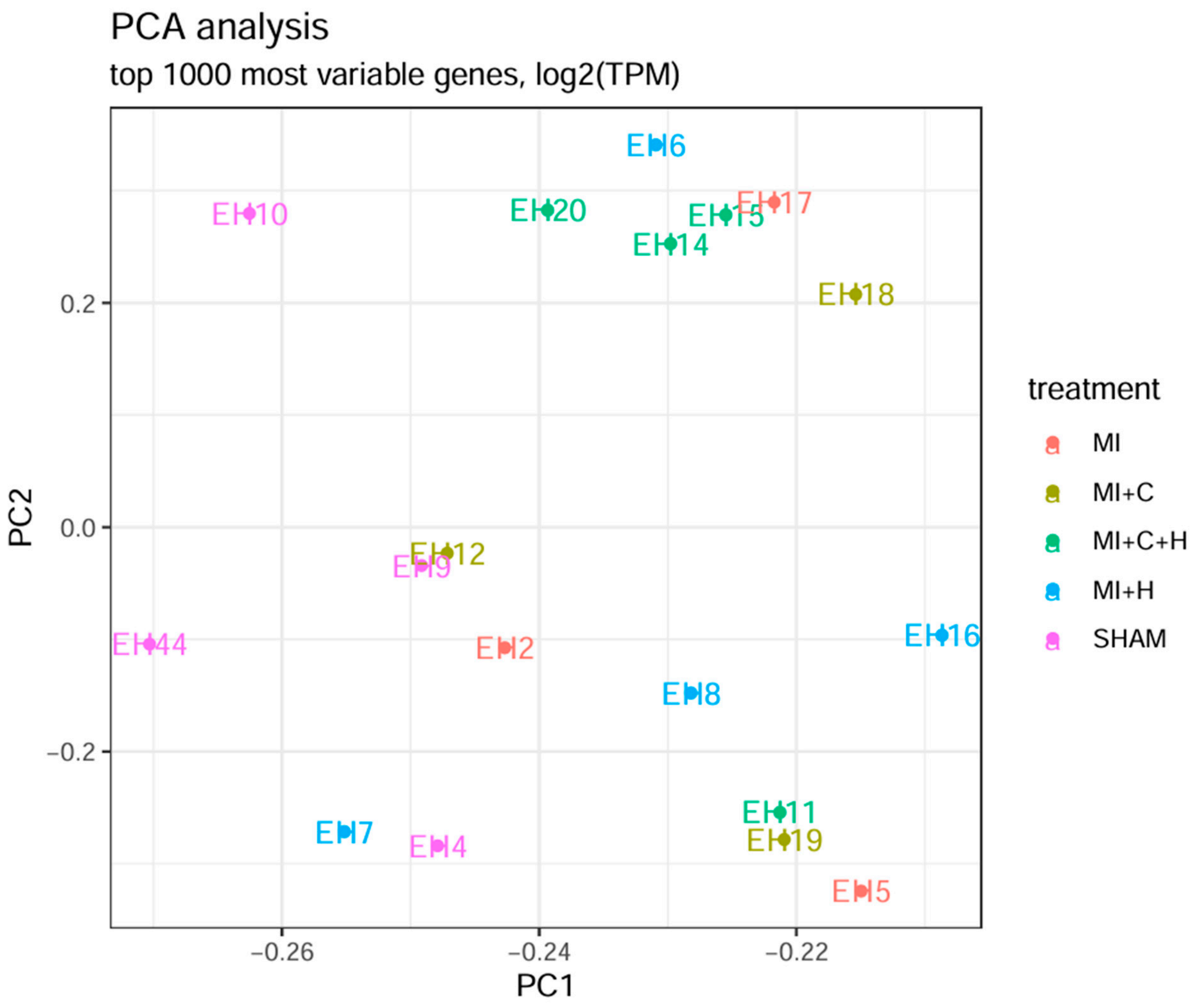

Supplementary figure S5. PCA plot of the included samples for RNAseq analysis. Samples were sequenced in one batch, where no clear clustering was observed. A total of 18 samples were included for the PCA analysis. Sample sizes per group were; SHAM: n = 4, MI: n = 3, MI+C: n= 3, MI+H: n = 4, MI+C+H: n = 4.
